# Spatiotemporal quantitative microRNA-155 imaging reports immune-mediated changes in a triple-negative breast cancer model

**DOI:** 10.3389/fimmu.2023.1180233

**Published:** 2023-06-08

**Authors:** Elena Skourti, Alessia Volpe, Cameron Lang, Preeth Johnson, Fani Panagaki, Gilbert O. Fruhwirth

**Affiliations:** ^1^ Imaging Therapies and Cancer Group, Comprehensive Cancer Centre, School of Cancer and Pharmaceutical Sciences, King’s College London, London, United Kingdom; ^2^ Department of Physics, King’s College London, London, United Kingdom

**Keywords:** cell tracking, macrophage, reporter gene imaging, sodium iodide symporter (NIS)/SLC5A5, triple-negative breast cancer, microRNA, tumor microenvironment, whole-body imaging

## Abstract

**Introduction:**

MicroRNAs are small non-coding RNAs and represent key players in physiology and disease. Aberrant microRNA expression is central to the development and progression of cancer, with various microRNAs proposed as potential cancer biomarkers and drug targets. There is a need to better understand dynamic microRNA expression changes as cancers progress and their tumor microenvironments evolve. Therefore, spatiotemporal and non-invasive *in vivo* microRNA quantification in tumor models would be highly beneficial.

**Methods:**

We developed an *in vivo* microRNA detector platform in which the obtained signals are positively correlated to microRNA presence, and which permitted stable expression in cancer cells as needed for long-term experimentation in tumor biology. It exploits a radionuclide-fluorescence dual-reporter for quantitative *in vivo* imaging of a microRNA of choice by radionuclide tomography and fluorescence-based downstream ex vivo tissue analyses. We generated and characterized breast cancer cells stably expressing various microRNA detectors and validated them *in vitro*.

**Results:**

We found the microRNA detector platform to report on microRNA presence in cells specifically and accurately, which was independently confirmed by real-time PCR and through microRNA modulation. Moreover, we established various breast tumor models in animals with different levels of residual immune systems and observed microRNA detector read-outs by imaging. Applying the detector platform to the progression of a triple-negative breast cancer model, we found that miR-155 upregulation in corresponding tumors was dependent on macrophage presence in tumors, revealing immune-mediated phenotypic changes in these tumors as they progressed.

**Conclusion:**

While applied to immunooncology in this work, this multimodal *in vivo* microRNA detector platform will be useful whenever non-invasive quantification of spatiotemporal microRNA changes in living animals is of interest.

## 1 Introduction

MicroRNAs are small non-coding RNAs that are post-transcriptional regulators of gene expression. They function through complementary complete or partial binding to 3’ untranslated regions (3’UTR) of target messenger RNAs, whereby in most cases they are responsible for messenger RNA degradation or inhibition of translation (1). Aberrant microRNA expression was found to be central to development and progression of human cancers and to define tumor types, prognosis, and treatment response (2, 3). For example, miR-21 represents the most commonly upregulated microRNA and is linked with poor prognosis (4), while miR-155 is one of the most important microRNAs linked to tumor progression. MiR-155 displayed oncogenic features in blood and solid cancers while it targets and inhibits many genes involved in immune response, DNA damage response, hypoxia, inflammation, and tumorigenesis (5). MicroRNAs have been proposed as disease biomarkers and as drug target/candidate therapeutics (2, 5). Nevertheless, surprisingly little is known about individual microRNA dynamics *in vivo* as tumors progress. To investigate the roles of specific microRNAs during cancer progression in animal tumor models, it would be highly beneficial to quantify microRNA expression changes non-invasively, spatially resolved, and over the whole duration of tumor model growth and progression.

Various reporter gene technologies for microRNA expression were developed including single and dual-reporter systems for cell imaging, and bioluminescence- and radionuclide-based preclinical applications followed (6–9). These approaches relied on detection of signal loss upon microRNA expression and presented with severe limitations as the signal loss can also be due to unspecific regulations of the promoter driving reporter expression or cell death followed by reporter degradation. Consequently, methods to positively correlate microRNA presence with reporter signals were developed. These were based on repressor proteins under microRNA control, and repressors then controlling reporter expression (10, 11).

As rodents are optically opaque, all optical whole-body imaging techniques on this scale are severely affected by differential light absorption, scatter and poor depth penetration, precluding full 3D quantification (12). In contrast, radionuclide imaging including single-photon emission computed tomography (SPECT) and positron emission tomography (PET) provide quantitative 3D data at depth and better resolutions compared to preclinical optical whole-body techniques. A pool of radionuclide imaging-compatible reporters is available (13) with the sodium iodide symporter (NIS;(14)) being a well-studied reporter detectable by both SPECT and PET (15, 16). Moreover, dual-modality reporter approaches have been developed, to bridge *in vivo* whole-body with *ex vivo* tissue level information. We have previously established NIS-fluorescent protein fusion reporters, and their utility for sensitive multi-modal cell tracking across scales has been demonstrated in preclinical cancer models (17–19).

Here, we developed an *in vivo* microRNA imaging platform to enable the long-term investigation of microRNA expression dynamics by quantitative whole-body *in vivo* imaging in animal tumor models. We present *in vitro* and preclinical *in vivo* validation data in breast cancer models. We used the platform to investigate mir-155 dynamics in breast tumors models as a function of innate immune cell presence.

## 2 Methods

### 2.1 Reagents

Reagents were from Merck, New England Biolabs, Sigma-Aldrich, Thermo-Fisher or VWR unless otherwise stated. Tissue culture materials were from Corning, Sarstedt or TPP. [^99m^Tc] ${\text{TcO}}_{4}^{\;-}$ was generator-eluted (on-site King’s Health Partners’ Radiopharmacy) as sodium salt solution and used within two half-lives from the time of generator elution, *i.e.* 12h.

### 2.2 DNA constructs

Information regarding the generation of DNA constructs is detailed in **Supplementary Materials**.

### 2.3 Cells

HEK293T, HCC1954, MCF-7, MCF-10A, MDA-MB-231, MDA-MB-361 and MDA-MB-436 cells were purchased from ATCC and grown according to the supplier’s recommendations. Media were further supplemented with penicillin (100IU/mL) and streptomycin (50 μg/mL) as well as drugs for selection where relevant (see below). All cell lines were confirmed negative for mycoplasma (LookOut-Mycoplasma PCR Detection, Sigma) quarterly throughout the study.

### 2.4 Lentivirus production and transduction

Lentivirus production was performed using HEK293T cells as previously described (18); for a brief description, see **Supplemental Materials**. Cell selection started 24h post transduction and was performed using either neomycin (2 mg/mL), puromycin (2 μg/mL) for detector cells and/or blasticidin 4 μg/mL) for cell lines stably overexpressing microRNAs or miR-lockers.

### 2.5 Determination of microRNAs in cells

Total cellular RNA was extracted using the miRCURY RNA Isolation Kit-Cell&Plant (Exiqon) according to the manufacturer’s instructions. Isolated RNA was stored at -80° C after concentration and purity determination using a SpectrostarNano spectrophotometer (BMG Labtech). The integrity of each RNA sample was checked by agarose gel electrophoresis (0.8% (w/v) agarose/TAE gel). Prior to loading onto the agarose gel, samples were denaturated with 10% (v/v) formamide and heated at 70°C. cDNA was synthesized only from intact total RNA samples using the miRCURY LNA™ Universal RT microRNA PCR kit (Exiqon) according to the manufacturer’s instructions. For RT-PCR, Pick&Mix microRNA PCR Panel 96-well Ready-to-Use (Exiqon) plates were used together with LNA primers for the following micro RNAs: miR-155, miR-21a, miR-221 as well as miR-674a and miR-324 as control/housekeeping miRNAs. RT-PCR was performed using ExiLENT SYBR^®^ Green master mix (Exiqon) according to the manufacturer’s instructions using a StepOne Real Time PCR System (Applied Biosystems).

### 2.6 Cell treatments with 4-Isopropylbenzoic acid

Cell treatments with 4-Isopropylbenzoic acid (cumate) involved seeding the indicated cells at densities of 10^5^ cells/cm^2^ 24h prior to treatment. Cells were treated with cumate at a final concentration of 500 μM for 24h.

### 2.7 Cell treatments with miRNA inhibitors

MicroRNA Power Inhibitors for miR-21 (Exiqon; #4101754-001) and miR-scr (Exiqon; #199006-001) were transfected into cells stably expressing the miR-21 detector (231.NGR21) using Lipofectamine RNAiMAX (Invitrogen). Therefore, cells were seeded at 5x10^4^ cells/cm^2^ in 24-well plates (10^5^ cells/well) 24h prior to transfection using the manufacturer’s protocol.

### 2.8 Cell proliferation

10^3^ indicated cells were plated per well in 96-well plates each in 100 μL growth media (without any selection drugs) and incubated at 37°C and 5% CO_2_ for up to 4d (one plate per day). On each day, Alamar Blue was added to a final concentration of 44 µM and plates were incubated for 2h at 37°C and 5% CO_2_. Individual plates were analyzed for fluorescence (excitation: 530 nm; emission: 580 nm) at each indicated time point; values were background corrected using wells with growth medium but containing no cells.

### 2.9 Determination of NIS function by radiotracer uptake

Determination of NIS function by radiotracer uptake was assessed by [^99m^Tc] ${\text{TcO}}_{4}^{\;-}$ uptake (50kBq/mL per 10^6^ cells) as previously described (17).

### 2.10 Fluorescence microscopy of cells

Indicated cells were seeded at 6x10^4^ cells/cm^2^ onto sterile acid-washed glass coverslips. Cells were cultured overnight in fully supplemented growth medium and then fixed with 4% paraformaldehyde (PFA) in PBS for 8 min and washed twice in PBS. Cells were then stained for DNA with Hoechst 33342 (1 µg/mL in PBS; 15 min at room temperature) before being washed twice with PBS, rinsed in deionized water, and mounted onto microscope slides using Mowiol-488 containing 2.5% (w/v) DABCO. Samples were dried overnight in the dark at room temperature and stored in the fridge until imaged on a Nikon Eclipse Ti2 wide-field fluorescence microscope equipped with the following filter sets (ex/em; all BP) for imaging Hoechst 33342 (AT350/50x; T400lp; ET460/50m), GFP/Alexa488 (ET470/40x; T495lpxr; ET525/50m), OrangeFISH#2/Cy3 (ET539/21x; T556lpxr; ET576/31m) and Cy5/Alexa647 (ET640/30x; T660lpxr; ET690/50m). Intensity-based image analysis involved thresholding based on negative control conditions as baseline; this was performed using identical image-processing workflows for images shown within the same figures and all associated quantifications. ImageJ software v1.53 was used to perform these analyses.

### 2.11 Flow cytometry of cells

Flow cytometry of cells was performed using live cells. Cells were washed twice with Hank’s buffered saline (HBSS with Ca^2+^ and Mg^2+^) supplemented with 1%(v/v) FCS/5mM EDTA to reduce cell aggregation. NIS-GFP expression was assessed by GFP fluorescence using a BD FACSCalibur flow cytometer (BD Bioscience) and analyzed using Flowing Software v2.5.1 (Turku Centre for Biotechnology).

### 2.12 Immunoblotting

Immunoblotting was performed as described before (20) using the following primary antibodies: polyclonal rabbit anti-GFP (Life Technologies; #MP11122, 1 μg/mL) and monoclonal mouse anti-GAPDH (Genetex, Taiwan R.O.C.; clone GT239, 0.5 μg/mL).

### 2.13 Ethics approval for animal work

All experimental protocols were monitored and approved by the King’s College London Animal Welfare and Ethical Review Body Animal Welfare and Ethical Review Panel, in accordance with UK Home Office regulations (PPL-70/8879) under the Animals (Scientific Procedures) Act 1986 and UK National Cancer Research Institute (NCRI) Guidelines for the Welfare and Use of Animals in Cancer Research.

### 2.14 Animal strains

NOD.Cg-Prkdc^scid^Il2rg^tm1Wjl^/SzJ (NSG) and CB17/Icr-Prkdc^scid^/IcrIcoCrl (SCID) mice were purchased from Charles River UK.

### 2.15 Animal husbandry and anaesthesia

All mice used were female and between 6 and 8 weeks old at the beginning of the experiment. Mice were maintained within the King’s College London Biological Services Unit under specific pathogen-free conditions in a dedicated and licensed air-conditioned animal room (at 23 ± 2°C and 40-60% relative humidity) under light/dark cycles lasting 12h every day. They were kept in individually ventilated standard plastic cages (IVC; 501cm^2^ floor space; from Tecniplast) including environmental enrichment and bedding material in the form of sterilized wood chips, paper stripes and one cardboard roll per cage. Maximum cage occupancy was five animals, and animals were moved to fresh cages with fresh environmental enrichment and bedding material twice per week. Sterilized tap water and food were available ad libitum; food was PicoLab Rodent Diet 20 (LabDiet) in the form of 2.5x1.6x1.0 cm oval pellets that were supplied at the top of the cages. For imaging, animals were anesthetized using isoflurane (1.5% (v/v) in pure O_2_). After imaging, mice were either left to recover from anesthesia (by withdrawal of anesthetic) in a pre-warmed chamber or sacrificed under anesthesia by cervical dislocation. No adverse events were associated with the procedures performed in this study and animals put on weight in line with strain expectations (data from Charles RiverUK) throughout. Sentinel animals were kept on the same IVC racks as experimental animals and confirmed healthy after completion of the studies.

### 2.16 Animal tumor models

Young adult (5-6 weeks-old) female SCID or NSG mice were used to establish the orthotopic mammary tumors (10^6^ cells injected into the mammary fat pad) from the indicated MDA-MB-231-derived cell lines. Therefore, cells were trypsinized, washed with pre-warmed Hank’s buffered saline without Ca^2+^ and Mg^2+^ (HBSS), re-suspended in HBSS and counted. Aliquots of 10^6^ cells in 50µL HBSS were injected into the mammary fat pad. Once palpable, tumor volumes were measured with calipers using the formula V = π/6·L·W·D, wherein L is length, W is width and D is depth of the palpable tumor. Tumor volumes were determined by qualified staff using calipers at least every third day throughout the study.

### 2.17 *In vivo* imaging and image analysis

Mice were anesthetized with 2%(v/v) isoflurane/O_2_. Intravenous injection of 30MBq [^99m^Tc] ${\text{TcO}}_{4}^{\;-}$ (in 100µL sterile PBS) was performed prior to SPECT/CT imaging (NanoSPECT/CT-Silver-Upgrade; Mediso) and was repeated on day 41 and 71 post tumor establishment. Animals were placed anaesthetized onto scanner beds and CT was performed (55kVp tube voltage, 1200ms exposure time, 360 projections). SPECT imaging was started 40min after radiotracer administration and lasted 30min for each image acquisition. Data were reconstructed using Tera-Tomo (Mediso) including corrections for attenuation, detector dead time, and radioisotope decay. Images were analyzed using VivoQuant software (inviCRO) enabling the delineation of volumes of interest (VOIs) for quantification of radioactivity. CT images were used for anatomical reference to draw VOIs within which radioactivity was determined. The total activity in the whole animal (tail excluded) at the time of tracer administration was defined as the injected dose (ID). Data were expressed as %ID/mL.

### 2.18 *Ex vivo* tissue analyses

All *ex vivo* analyses are from harvested tumors collected at the end of indicated *in vivo* experiments. To record whole-tumor fluorescence a Fluorescence-labelled Organism Bio-imaging Instrument was used (NEOscience).

For tissue radioactivity analysis, animals were culled for tissue harvesting 75 min after administration of 20 MBq [^99m^Tc] ${\text{TcO}}_{4}^{\;-}$ in saline. Harvested tissues were weighed, and radioactivity was quantified using a γ-counter (1282-Compugamma/LKB-Wallac), alongside ^99m^Tc calibration standards. Data were expressed as standard uptake value (SUV).

For histology, excised tumors were embedded in optimal cutting temperature medium (OCT), frozen in liquid nitrogen vapor and stored at -80C until cryo-sectioning. 5 µm thick sections were cut and fixed in 4% paraformaldehyde (PFA) in PBS for 10 min, permeabilized (0.2% (v/v) Triton X-100 in PBS) and washed twice in PBS. Subsequently, sections were subjected to blocking buffer (PBS containing 2% (v/v) donkey serum, 1% (v/v) mouse serum, 2% bovine serum albumin (BSA) and 0.1% (v/v) Triton X-100) before being incubated overnight at 4°C with the indicated primary antibodies diluted in blocking buffer; antibodies used at 1-2 µg/mL were: anti-GFP (monoclonal rat; [D153-3] from MBL or polyclonal rabbit #MP11122 from Invitrogen); anti-F4/80 (monoclonal mouse; [BM8] from BMA Biomedicals); human-CK-18 (polyclonal sheep; AF7619m from R&D Systems). After two PBS washes, sections were stained with corresponding secondary antibody solutions in blocking buffer for 45 min at room temperature and in the dark; secondary antibodies used at 1 µg/mL were: donkey anti-rabbit conjugated to Cy5 (#711-175-152), donkey anti-rat conjugated to Cy2 (#712-225-153), donkey anti-mouse conjugated to Cy3 (#715-165-151), donkey anti-sheep conjugated to AlexaFluor647 (#713-605-147) and all were AffiniPure IgG (H+L) products from Jackson ImmunoResearch. Next, sections were stained for DNA with Hoechst 33342 (1 µg/mL in PBS; 15 min at room temperature) before being washed twice with PBS, rinsed in deionized water, and mounted onto microscope slides using Mowiol-488 containing 2.5% (w/v) DABCO. All solutions were sterile filtered (0.2 µm pores) before use. Samples were dried overnight in the dark at room temperature and stored in the fridge until imaged on a Nikon Eclipse Ti2 wide-field fluorescence microscope equipped with the following filter sets (excitation, dichroic, emission filters) for imaging Hoechst 33342 (AT350/50x; T400lp; ET460/50m), GFP/Alexa488 (ET470/40x; T495lpxr; ET525/50m), OrangeFISH#2/Cy3 (ET539/21x; T556lpxr; ET576/31m) and Cy5/Alexa647 (ET640/30x; T660lpxr; ET690/50m). Fiji/ImageJ v1.5x was used for image processing. CellProfiler v3.1.9 was used for the automated segmentation of different cell types identified by immunofluorescence imaging. Therefore, a pipeline was created that identified individual cells and distinguished clumped cells based on image staining intensity and cell morphology to determine the number of F4/80^+^, NIS-GFP^+^, DAPI^+^ and CK-18^+^ objects/cells per image. %NIS-GFP^+^CK-18^+^ and F4/80^+^ cells per total cells were calculated and presented as cumulative data (see **Supplementary Materials** for a segmentation workflow example).

### 2.19 Statistical analyses

Statistical analyses were done by GraphPad Prism v8.x with statistical test details in figure legends.

## 3 Results

### 3.1 Construction and *in vitro* assessment of the microRNA detector

**Figure 1:**
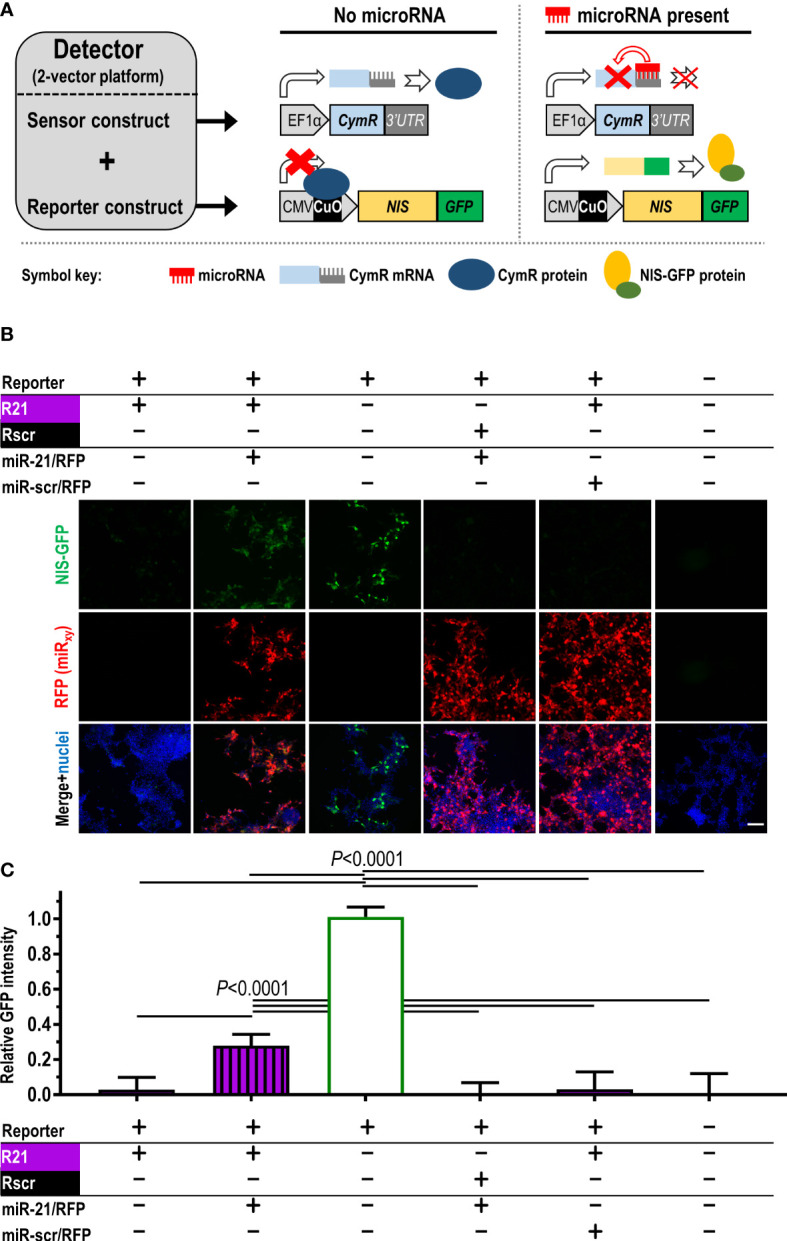
Construction and validation of the microRNA detector employing the dual-vector platform. **(A)** Scheme detailing detector components and function. Constitutively (EF1α promoter) driven CymR repressor forms the sensor, which is controlled *via* its 3’UTR by the microRNA of interest. CymR protein regulates expression of the imaging reporter NIS-GFP by interaction with the CuO operator that is part of the CMV/CuO promoter driving the reporter. Upon microRNA presence, CymR expression is inhibited followed by the CMV/CuO promoter gaining function and subsequent expression of the of NIS-GFP reporter. **(B)** Validation of the detector principle in HEK293T cells using the dual-vector platform. The sensor is either under the control of miR-21 (R21, purple) or a non-targeting scrambled microRNA (Rscr, black). Validation included microRNA co-expression (miR-21 or miR-scr) from separate plasmids (detectable by RFP expression) as indicated. Signals are only recorded when the correct microRNA matching the reporter is expressed (compare columns 1,2, and 5). Controls include column 3 (no repressor), vice-versa non-matching sensor/microRNA conditions (columns 4), and no detector system at all (column 6). Typical results shown; scale bar=100μm. **(C)** Cumulative results of **(B)**; *n*≥5, error bar=SD. *P*-values by one-way ANOVA with Tukey’s multiple comparison correction.

Our aim was to generate an *in vivo* imaging-compatible detector platform that reports on microRNA changes dynamically, *via* signals that are positively correlated to microRNA concentrations, and that performs reliably when stably expressed in cancer cells. Adopting a repressor-based strategy, we exploited the cumate-controlled operator in its repressor configuration for mammalian use (CymR; (21)) and placed it under the control of a specific 3’UTR matching the microRNA of interest. In the absence of the microRNA of interest, this was intended to result in repressor expression and consequently no signal from the reporter, which was placed under transcriptional repressor control (**Figure 1A**). As reporter components of the detector, we used (i) NIS to enable non-invasive quantitative 3D imaging and (ii) fused NIS to monomeric green fluorescent protein to streamline cell production, *in vitro* validation in cells, and *ex vivo* tissue analyses (NIS-GFP; (17)). Both constructs were delivered into cells separately (‘dual-vector’ system; see also further below). The detector was first validated *in vitro* using the miR-21 in HEK293T cells as a model because these cells expressed negligible miR-21 levels (22–24). Upon expression of the sensor plasmid R21 (responsive to miR-21), NIS-GFP reporter expression was undetectable in HEK293T cells (**Figures 1B, C**/column 1) as was expected. When co-expressing miR-21 in HEK293T cells alongside the miR-21 detector, signal repression was released and NIS-GFP was expressed (**Figures 1B, C**/column 2). In the absence of a sensor plasmid (positive signal control), the NIS-GFP reporter was always highly expressed (**Figures 1B, C**/column 3). This data confirmed the function of the platform as described in **Figure 1A**.

To test specificity for a chosen microRNA, we performed the following experiments: (i) co-expression of a mismatch microRNA for the repressor under miR-21 control (R21), *i.e.* the scrambled microRNA miR-scr (designed to not have any predicted targets in human, rat and mouse), which resulted in reporter repression and no NIS-GFP signals (**Figures 1B, C**/column 4); (ii) the reverse scenario, in which the repressor was placed under the control of a 3’UTR matching miR-scr (Rscr) paired with miR-21 overexpression, which also did not result in NIS-GFP signals differing from background levels (**Figures 1B, C**/column 5). This demonstrated the specificity of the platform, which was conferred by the individual 3’UTRs.

Notably, a ‘single-vector’ configuration appeared attractive to us, because it might have simplified stable cell line generation. As issues were reported before with self-contained bidirectional designs (10), we opted for a multi-cistronic unidirectional design (**Figure S1A**). However, using this single-vector platform in experiments analogous to **Figure 1** demonstrated insufficient release of repression upon co-expression of matching microRNAs of interest (**Figures S1B, C**). Consequently, we continued all further experimentation with the ‘dual-vector’ configuration.

### 3.2 Establishment and *in vitro* validation of stable microRNA detector expressing breast cancer cells

We next quantified the cellular amounts of miR-21, miR-221 and miR-155, which have previously been reported to be involved in tumor growth, progression and metastasis, in several well-established breast cancer cell lines; they included HCC1954 (ER^-^, PR^-^, HER2^+^), MDA-MB-231, MDA-MB-436 (both triple-negative), MDA-MB-361 (ER^+^, PR^weak^, HER2^+^) and MCF-7 (ER^+^, PR^+^, HER2^-^) (25) as well as pre-cancerous MCF-10A cells (**Figure S2**). MiR-21 and miR-221 differed largely between the cell lines, and miR-155 expression was not detected in any of them despite its known oncogenic role in breast cancer (26, 27). We chose MDA-MB-231 as a well-established triple-negative breast tumor model for further experimentation (28, 29).

**Figure 2:**
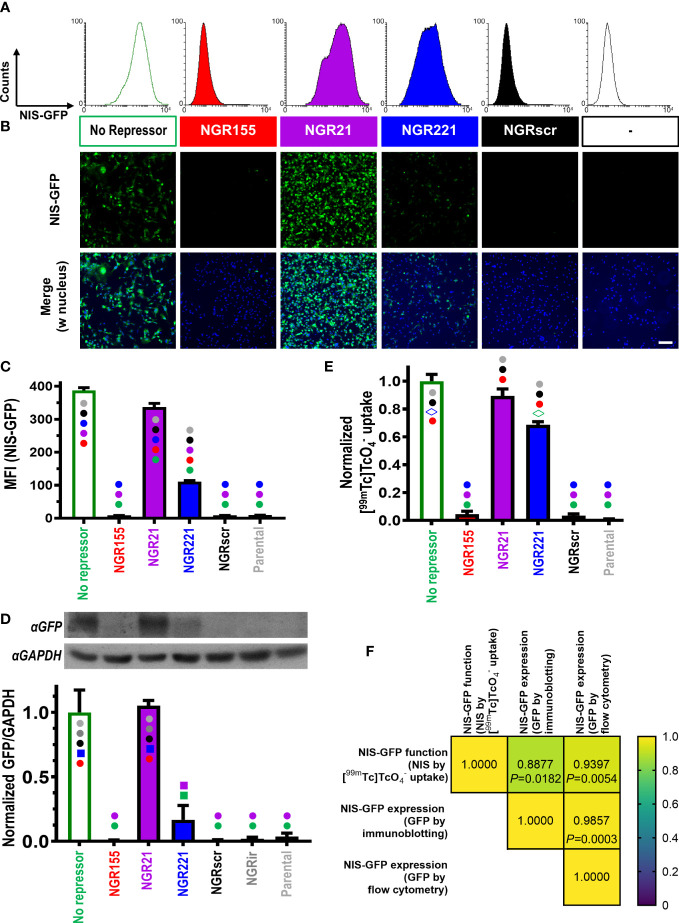
Characterization of MDA-MB-231 cells stably expressing different microRNA detectors. Representative **(A)** flow cytometry histograms and **(B)** fluorescence micrographs of indicated microRNA detector-expressing cells (scale bar=100μm). **(C)** Cumulative mean fluorescence intensity (MFI) data of cells in **(A)**; *n*=3, error bar=SD. **(D)** (*Top*) Representative immunoblots and (*bottom*) cumulative data of corresponding densitometry; ‘NGRir’ represents a different negative control cell line, which expresses another detector type intended to pick up a microRNA that is designed not to be present in human cells. **(E)** Cumulative data of NIS radiotracer [^99m^Tc] ${\text{TcO}}_{4}^{\;-}$ uptake by γ-counting of cell pellets. For both (D/E) data is normalized to the positive control NIS-GFP without any repressor present; *n*=3; error bars=SD. (C/D/E) Statistical analysis is based on one-way ANOVA including Tukey’s multiple-comparison corrections yielding *P*-values. Significant differences between cell lines are displayed as follows: cell lines are marked with different symbols in varying colors. Symbol type indicates the level of significance (●: *P*<0.0001; ■: *P*<0.001; ◊: *P*<0.01). Symbol colors indicate for each cell line the individual comparisons for which the significance level indicated by the symbol has been reached. Comparisons that are not significant are reflected by no symbol being present on a given column. **(F)** Correlation matrix for pairwise Pearson correlations of NIS-GFP reporter expression **(C, D)** and NIS-GFP reporter function **(E)**: (*Top*) Pearson Cross correlation coefficients and (*bottom*) corresponding *P*-values.

For animal tumor models, it was paramount to use ensure stable detector-expression and thereby enable long-term *in vivo* experimentation. Hence, MDA-MB-231 cells (231) were lentivirally transduced with the ‘dual-vector’ detector platforms and selected to generate the following stable cell lines: 231.NGR155 (miR-155 detector), 231.NGR21 (miR-21 detector), 231.NGR221 (miR-221 detector), 231.NGRscr (detector for scrambled control microRNA), and 231.NG (reporter but no repressor expressed, thus serving as maximum positive control for reporter signals). Stable cell lines were analyzed by flow cytometry, fluorescence microscopy and immunoblotting (**Figures 2A–D**), all of which demonstrated reporter signals to be in line with microRNA levels determined for parental MDA-MB-231 cells. For example, we did not observe reporter signals in 231.NGR155 cells (**Figures 2**/red) in line with miR-155 being undetectable in parental MDA-MB-231 cells (**Figure S2**). Moreover, we found that 231.NGR21 and 231.NGR221 cells both produced reporter signals (**Figures 2**/purple and blue, respectively), which was also in agreement with cellular microRNA quantification (**Figure S2**). Importantly, the function of the radionuclide reporter NIS, as determined by cellular uptake of the radiotracer [^99m^Tc] ${\text{TcO}}_{4}^{\;-}$ , correlated well with reporter expression as determined by GFP-based assays (**Figure 2F**). This is notable as the reporter function assay involves signal amplification (one reporter taking up several radioisotopes) and requires correct intracellular localization of NIS-GFP in the cellular plasma membrane to function, while GFP-based assays rely merely on reporter presence without discrimination of subcellular localization. Together, this data showed that the detectors reported cellular microRNA levels with both radionuclide and fluorescence read-outs, which well agreed with qPCR-determined cellular microRNA amounts (**Figure S2**). Notably, we observed signal levels in 231.NGRscr cells to be the same as background levels, which revealed that the detector specific for the scrambled microRNA did not produce false-positive signals (**Figure 2**).

The CymR repressor can also be pharmacologically released from its DNA binding site. In otherwise dark 231.NGR155 cells, we observed NIS-GFP signals when we treated them with cumate demonstrating pharmacological de-repression and thus specificity for the repressor (**Figure S3**).

### 3.3 MicroRNA modulation in stable microRNA detector-expressing cells

**Figure 3:**
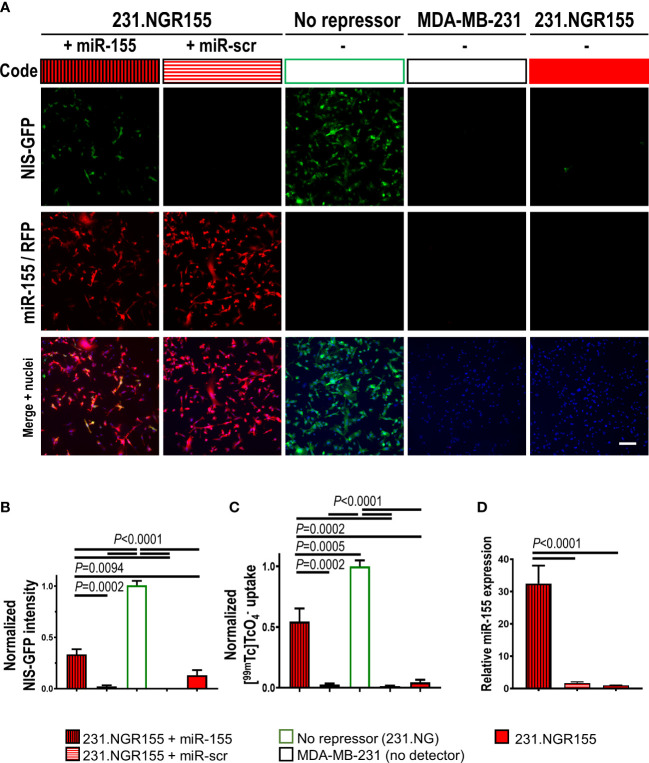
Validation of miR-155 response in miR-155 detector-expressing MDA-MB-231 cells. **(A)** Representative fluorescence images of cells expressing indicated microRNA detectors and co-expressing indicated microRNAs. Scale bar=100μm. **(B)** Cumulative intensity-based NIS-GFP fluorescence quantification by microscopy. **(C)** Cumulative data of NIS radiotracer [^99m^Tc] ${\text{TcO}}_{4}^{\;-}$ uptake as determined by γ-counting of cell pellets. **(D)** Quantitative RT-PCR-based analysis of miR-155 amounts in indicated cells. Data is normalized to the positive control NIS-GFP without any repressor present; *n*=3; error bars=SD. *P*-values by one-way ANOVA including Tukey’s multiple-comparison corrections.

Next, we determined how 231.NGR155 cells responded to overexpression of different microRNAs to investigate specificity in response to different microRNAs. Overexpression of miR-155 in 231.NGR155 cells resulted in NIS-GFP signals as detected by GFP fluorescence (**Figures 3A, B**, protein-level), NIS radiotracer uptake (**Figure 3C**, reporter function), and qPCR reporting on cellular miR-155 levels (**Figure 3D**). Notably, overexpression of a scrambled microRNA, did not change any baseline signals.

Moreover, we assessed whether our detector platform would respond also to partial reduction of an endogenous microRNA in stable detector-expressing cells. Therefore, we either co-expressed antagonistic miR-21 (miR-21-locker) in 231.NGR21 cells or treated these cells with a miR-21 inhibitor. In both cases we found significantly lower reporter signals in treated cells compared to vehicle-treated 231.NGR21 cells (**Figure S4**).

### 3.4 *In vivo* validation of microRNA detector-expressing tumors in immunodeficient mice

**Figure 4:**
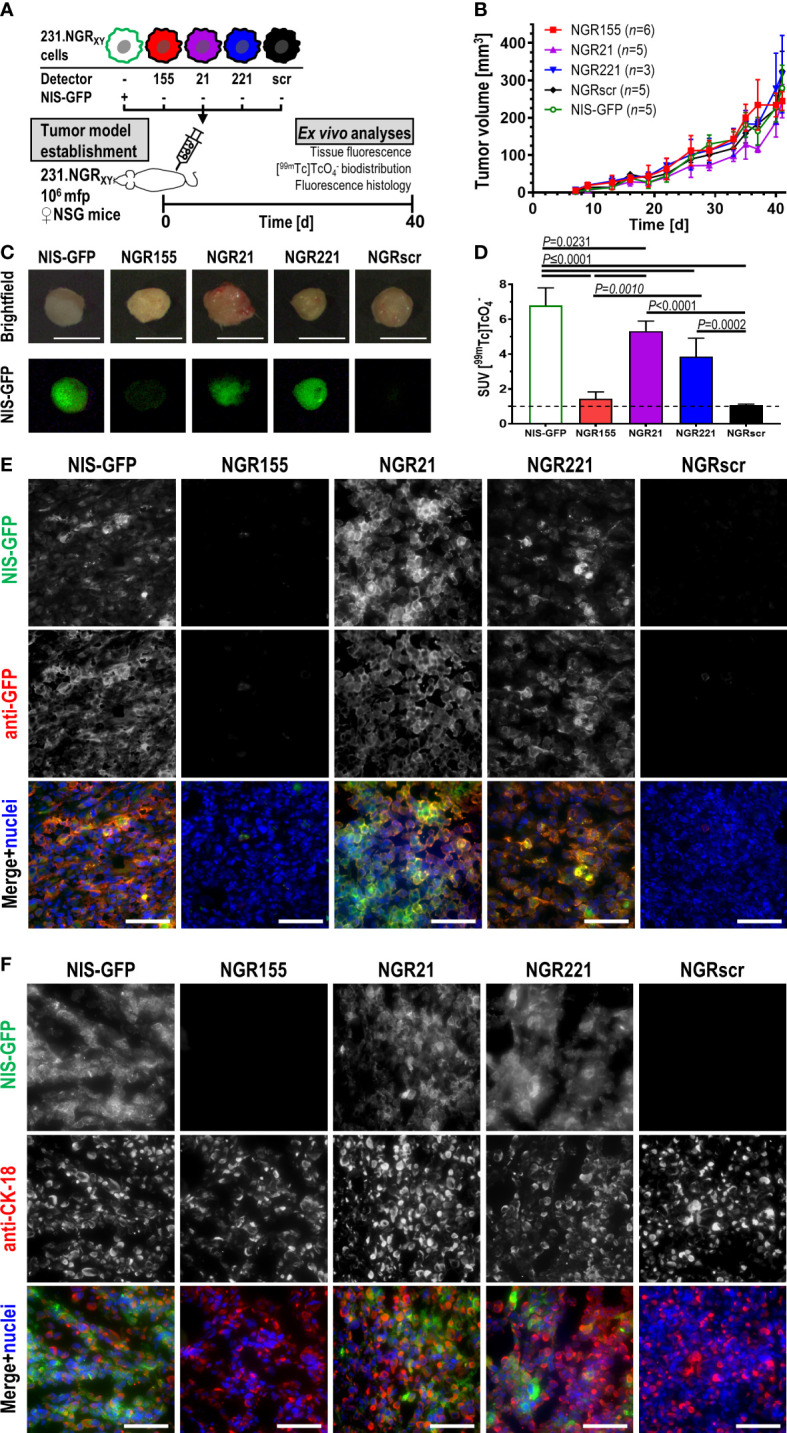
*In vivo* validation of microRNA detector expressing MDA-MB-231 cell lines in immunodeficient NSG mice. **(A)** Experimental scheme. **(B)** Tumor growth curves of indicated tumor models. Cumulative data are shown (for *n* see inset); error bars=SD. Repeated measures mixed-effect analysis including Geisser-Greenhouse correction comparing simple effect within time points using Tukey’s multiple comparison correction revealed no significant differences between tumor cohorts (*P*>0.05). On day 40, animals received 20 MBq of the NIS radiotracer [^99m^Tc] ${\text{TcO}}_{4}^{\;-}$ and were culled 75 min later with tumors harvested and analyzed immediately for their **(C)** tissue fluorescence and **(D)** tissue radioactivity by γ-counting. **(C)** Typical examples of harvested tumors are shown. Brightfield images were taken under room light, while fluorescence images were taken using blue light LED excitation and through a green light filter in the dark; scale bars are 1 cm. **(D)** Cumulative data of tumor radioactivity are shown and expressed as standard uptake value (SUV); for *n* see inset of **(B)**, error bars=SD. One-way ANOVA with Tukey’s multiple comparisons correction revealed NGR155 tumors did not differ from NGRscr tumors while all other tumors showing radiotracer uptake significantly above of both NGR155 and NGRscr tumors. **(E, F)** Typical immunofluorescence histology micrographs of tumor sections cut from tumors in **(C, D)** and stained for the indicated antibodies. In merged images, NIS-GFP is pseudocolored in green, anti-GFP staining in **(E)** and anti-CK-18 staining in **(F)** are pseudocolored in red, and stained nuclei in blue; scale bares are 100 μm.

Stable microRNA detector-expressing 231 cell lines were found to proliferate *in vitro* like parental cells (**Figure S5**). We used them to establish orthotopic xenograft tumors in immunodeficient female *NOD.Cg-Prkdc^scid^ Il2rg^tm1Wjl^/SzJ* (NSG) mice and investigated tumor tissues after 40 days when sizeable tumors had formed (**Figures 4A, B**). Animals received the NIS radiotracer [^99m^Tc] ${\text{TcO}}_{4}^{\;-}$ and 75 min later tumor tissues were harvested for tissue radioactivity determination and tissue fluorescence imaging. Tumor fluorescence was observed in 231.NGR21, 231.NGR221 and positive control 231.NG tumors while only background tissue autofluorescence was observed in 231.NGR155 and NGRscr tumors (**Figure 4C**). These results were in line with *ex vivo* tissue radioactivity data from these harvested tissues which reported on radiotracer uptake due to the activity of the NIS reporter portion (**Figure 4D**). Moreover, tumor histology confirmed this pattern as tissue immunofluorescence microscopy revealed NIS-GFP reporter signals in 231.NGR21, 231.NGR221 and 231.NG tumors (**Figure 4E**; *via* staining with an anti-GFP antibody). Notably, there we found NIS-GFP localized to plasma membranes of cancer cells also *in vivo*, which is a prerequisite for correct function of the NIS reporter portion and therefore radiotracer uptake. We also confirmed that NIS-GFP expressing cells were indeed human cancer cells through staining with anti-human cytokeratin-18 (CK-18) (**Figure 4F**). We also provide comparative growth and *ex vivo* radionuclide-fluorescence reporter data from tumors established using parental MDA-MB-231 cells (**Figure S6**). Next, we established 231.NGR155 tumors from cells that were overexpressing either miR-155 or the scrambled miR-scr. Results obtained were in line with our previous observations in cell lines (**Figure S7**). Data were in line with above-described *in vitro* data and showed not only that detector expression was stable over weeks in animal tumor models, but also that the relative features observed in cell lines were retained *in vivo* in the corresponding tumors and that the detector-expressing cancer cells were detectable both macroscopically and microscopically.

### 3.5 *In vivo* imaging of microRNA-detectors in mice with residual innate immunity

**Figure 5:**
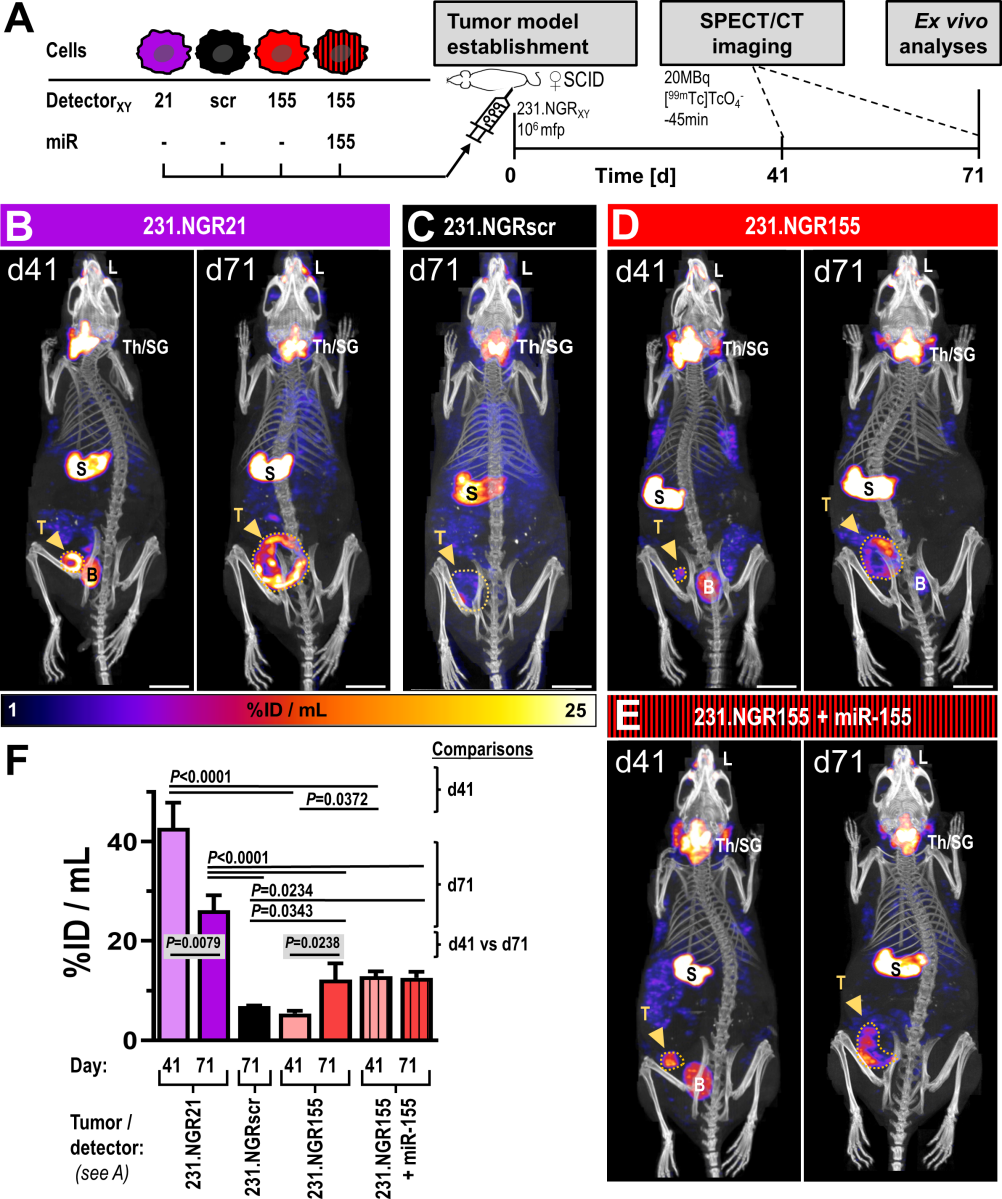
*In vivo* SPECT/CT imaging of detector-expressing tumor models in SCID mice. **(A)** Experimental scheme. Representative images of tumor models **(B)** 231.NGR21, **(C)** 231.NGRscr, **(D)** 231.NGR155 and **(E)** 231.NGR155 co-expressing miR-155 imaged by [^99m^Tc] ${\text{TcO}}_{4}^{\;-}$ afforded NIS-SPECT/CT at indicated times after tumor model establishment. Images are maximum intensity projections (MIP) of reconstructed SPECT images (hue) and overlaid by CT images providing anatomical information (grayscale). Endogenously NIS expressing organs (thyroid/salivary glands (Th/SG), stomach (S), lacrimal glands (L)) are visible alongside specific miR-expressing cancer cell signals in tumors (dark-yellow arrows). Notably, only living tumor cells that also express the respective microRNAs are detected. Bladder signals **(B)** stem from radiotracer excretion. Widest projected tumor margins from CT (dark-yellow dotted lines). Scale bars are 1 cm. **(F)** Cumulative SPECT analysis of radiotracer uptake in the tumors. *N*=3 as three mice were imaged per cohort, error bars=SD; *P-*values for comparisons between different cohorts on day 41 or day 71 were results of analyses by one-way ANOVA using Tukey’s multiple-comparison correction. Comparisons between different time points of the same cohort were performed using two-tailed Student’s *t*-test. Comparisons that were not significant are not visualized.

Next, we investigated whether the presence of functional innate immune cells might affect miR-155 expression in tumors using our MDA-MB-231-derived detector cell lines. Therefore, we employed female *CB17/Icr-Prkdc^scid^/IcrIcoCrl* (SCID) mice, which present with largely intact innate immunity including functional monocytes/macrophages, dendritic cells and NK cells but lack adaptive immunity (no T- and B-cells). Here, we also employed non-invasive repeat-*in vivo* imaging of detector signals by SPECT/CT using the NIS radiotracer [^99m^Tc] ${\text{TcO}}_{4}^{\;-}$ (**Figure 5A**). As expected, these tumor models in SCID mice grew slower and with larger variation (**Figure S8**) compared to those previously established in immunodeficient NSG mice (**Figure 4B**). In tumors established from 231.NGR21 cells (detector positive control as miR-21 is expressed in these cells), we recorded strong NIS-GFP signals in tumors (**Figure 5B**/purple) both after 41 and 71 days. At day 71, SPECT imaging revealed areas within tumor volumes that lacked NIS signals. These areas indicated regions in which tumor cells had died as NIS imaging depends on an intact Na^+^/K^+^ gradient thereby enabling exclusive quantification of live cancer cells but not necrotic areas (19, 30). Other signals not stemming from tumor cells were also observed in these mice; they belonged to organs either endogenously expressing mouse NIS (*i.e.* thyroid and salivary glands, stomach, lacrimal glands) or were part of the renal radiotracer excretion route (*i.e.* bladder). Importantly, none of these organs/signals interfered with the objectives of this study. In 231.NGRscr tumors (**Figure 5C**/black; detector negative control), we did not observe any NIS signals stemming from tumor cells, in line with expectations from *in vitro* experiments.

**Figure 6:**
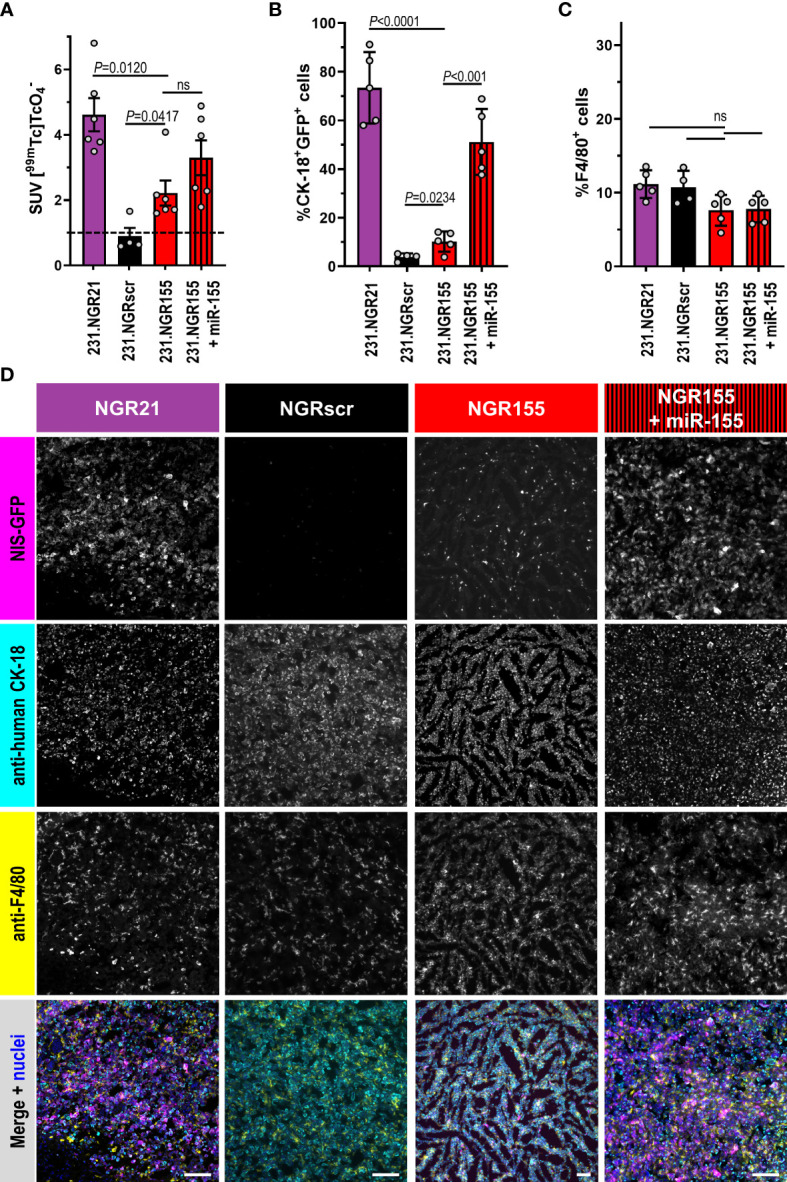
*Ex vivo* tissue analyses of microRNA detector expressing tumors grown in SCID mice. Indicated tumor models in SCID mice culled 75 min after radiotracer administration and *in vivo* imaging (**Figure 5**) were **(A)** analyzed for tissue radioactivity (*n*=6/4/6/6; for other organs see **Figure S9**); error bars=SEM. **(B)** Representative fluorescence histology (*n*=5/4/5/5; scale bars=100μm) quantitatively analyzed by CellProfiler for percentage of **(C)** CK-18^+^NIS-GFP^+^ cells (tumor cells reporting miR presence) or **(D)** F4/80^+^ macrophages per total cells in tumor tissues (100% represents all cells in the tissue including cancer, immune, and stromal cells). Indicated cell types in tissues were identified by anti-human CK-18 or anti-F4/80 staining with total cell numbers determined from staining nuclei with Hoechst 33342. In merged images, NIS-GFP fluorescence is pseudocolored in magenta, anti-CK-18 staining in cyan, anti-F4/80 in yellow, and stained nuclei in blue. Error bars=SD. In **(C, D)**, grey circles represent individual mice (each an average of *n_Tissue_* =5 different non-adjacent tissue sections/tumor). *P*-values by one-way ANOVA with Tukey’s multiple comparison correction. ns, not significant.

Surprisingly, we recorded small patches of radioactivity in different parts of 231.NGR155 tumors at day 41 (**Figure 5D**/red). After 71 days, these tumors had grown and the regions in which we recorded NIS signals became much more widespread within these tumors. These tumors were not uniformly showing detector signals most likely due to the onset of necrosis and the intrinsic detection of only live tumor cells at late time point (*cf.* above). In additional control tumors established from 231.NGR155 cells that co-expressed miR-155 (**Figure 5E**/red-black stripes), we found the expected radioactivity uptake throughout the tumor (bar necrotic areas) across both time points (*i.e.* similar to 231.NGR21 tumors; **Figure 5E**/B, respectively). Notably, radioactivity uptake in 231.NGR155 tumors was significantly smaller compared to 231.NGR155 tumors overexpressing miR-155 when assessed by imaging at the earlier day 41 time point (**Figure 5F**), which indicated less widespread miR-155 expression or lower miR-155 expression in the 231.NGR155 tumors compared to the maximum signals that were obtained when all cells express miR-155 (*i.e.* as in the 231.NGR155+miR-155 expression model). After culling animals on day 71, tissue radioactivity determined by γ-counting (**Figure 6A**) revealed significantly increased radioactivity amounts in 231.NGR21 tumors (detector positive control) compared to 231.NGRscr tumors (detector negative control) in line with expectations. Importantly, 231.NGR155 tumors also had significantly elevated radioactivity levels compared to 231.NGRscr tumors indicating the switch-on of the miR-155 detector in these tumors when grown in this mouse strain. Tumors formed from 231.NGR155+miR-155 cells showed similar results compared to 231.NGR155 tumors at this late time point, in agreement with our *in vivo* imaging results from day 71 (**Figure 5**).

Furthermore, employing immunofluorescence histology to analyze tissue composition expectedly showed 231.NGR21 tumors to contain large amounts of NIS-GFP-positive tumor cells (*i.e.* also positive for human CK-18; **Figures 6B, C**/purple). In line with previous observations in SCID mice, there were also many macrophages that had infiltrated the tumors at this late time point (detected by the established murine macrophage marker F4/80; **Figures B, D**). In contrast, 231.NGRscr negative control tumors contained no NIS-GFP-positive human tumor cells. Importantly, in 231.NGR155 tumors we also found NIS-GFP-expressing human tumor cells (by CK-18) and they formed >10% of the tumor mass in the analyzed sections. These were the tumor cells responsible for the radiotracer uptake as observed by *in vivo* imaging and *ex vivo* γ-counting (**Figures 5F**, **6A**, respectively). As expected, 231.NGR155 tumors co-expressing miR-155 showed higher amounts of NIS-GFP/CK-18 double-positive cancer cells in tumors than 231.NGR155 tumors. Notably, tissue sections across all tumor models were similarly infiltrated with F4/80-positive monocytes and macrophages (**Figures 6B, D**) indicating no negative impact of microRNA detector expression in the cancer cells on tumor infiltration by F4/80-positive cells. This *ex vivo* histology data independently confirmed our observations from *in vivo* imaging. Importantly, our combined data from applying the miR-155 detector to MDA-MB-231 tumors revealed distinct differences in miR-155 presence during progression in these tumors, which depended on the mouse strain (immunodeficient NSG mice *versus* partly immunocompromised SCID mice).

## 4 Discussion

Non-invasive long-term serial *in vivo* imaging of microRNA expression in animal tumor models has to the best of our knowledge not been done before. It requires stable detector expression and the use of radionuclide imaging to unlock quantitative 3D tomographic data. Here, we used human NIS, which can be used as an imaging reporter for both SPECT and PET, in human cells transplanted onto partly or fully immunocompromised mice. If a fully syngeneic murine model is desired in the future, human NIS can be swiftly replaced by murine NIS to ensure that the reporter would not elicit any immune response *per se* (31) with the option to also omit the fluorescent protein as needed. Detection limits for NIS-traceable cancer cells have previously been determined [~500-1000 cells/million cells: (17, 32)] and NIS signals rely on reporter function rather than mere reporter presence, which presents with the advantage of detecting living cells only (19).

More generally and across various fields, microRNA ‘signal-off’ approaches came with severe limitations, which is why a few ‘signal-on’ approaches were previously developed (10, 11). One such approach also included a radionuclide reporter (33), however the authors directly administered reporter plasmid DNA to animals without evaluation of reporter persistence or delivery/perfusion controls and in the context of muscular atrophy. None of the previous studies has focused on the needs of tumor biology as we did here. The question how to best introduce the detector platform stably into cancer cells crystallized around whether to employ a ‘single-vector’ or ‘dual-vector’ approach. While the first appeared more attractive due to simpler cell engineering, we found our ‘dual-vector’ approach to function as expected while our unidirectional ‘single-vector’ design failed to perform well (compare **Figure 1** with **Figure S1**). A different bidirectional ‘single-vector’ design was not considered for this work, because others had already reported significant problems with it (10). Consequently, we focused on our ‘dual-vector’ approach, and it enabled us to successfully generate a series of stable microRNA detector cell lines based on the human triple-negative breast cancer line MDA-MB-231. We successfully validated these microRNA detector-expressing cell lines *in vitro* using various independent methods whereby we found our detector platform to reliably report on expected microRNA levels (**Figures 2**, **3**, **S3, S4**). This was also confirmed *in vivo* using xenograft tumor models in immunodeficient NSG mice (**Figures 4**, **S7**). Importantly, these results demonstrated that our microRNA detector platform remained stable *in vivo* over several weeks in these tumor models and their corresponding microenvironments, which enabled their application to new microRNA imaging-informed research in the context of tumor progression.

As microRNAs were known to change cell phenotypes, we were interested in potential dynamic changes during tumor progression. MiR-155 had been implicated in breast cancer progression (26) but was not detected *in vitro* in MDA-MB-231 cells (**Figure S2**). Interestingly, using a mouse strain lacking adaptive but presenting with near-complete innate immunity (SCID), we found significant increases of miR-155 expression over time compared to control animals (**Figure 5**). All *ex vivo* analyses confirmed our *in vivo* data in SCID mice (**Figure 6**). Notably, in NSG mice lacking an adaptive immune system and NK cells, and which present with defective innate immunity associated with functionally immature macrophages and deficiencies in cytokine signaling (34), no miR-155 upregulation was found (**Figure 4**). This emphasized the role of functional monocyte/macrophage presence for the observed upregulation of miR-155 in the MDA-MB-231/SCID model. Our data is in line with a similar phenomenon identified by others who reported miR-155 upregulation in neuroblastoma cell lines as a consequence of macrophage infiltration and their ‘re-education’ to become tumor-promoting (35). These authors further identified miR-21 stemming from the neuroblastoma cells as the initial mediator of a cancer cell-macrophage communication loop with the messenger suggested to be exosomes with microRNA cargoes. Cancer-originated miR-21 was reported to trigger tumor-associated macrophages to shed miR-155-containing exosomes, which in turn were consumed by the cancer cells and caused miR-155 expression in these cancer cells (35, 36). It is therefore intriguing to hypothesize that the high miR-21 expression of our MDA-MB-231 model (which is known to produce extracellular vesicles/exosomes; (37, 38)) has been involved in such a cell-cell communication process in a similar manner as reported for neuroblastoma. However, as the purpose of this work was to establish dynamic and long-term *in vivo* microRNA imaging capability in tumor models, we refrained from further investigation of the precise cell-cell communication mechanisms involved in these particular triple-negative breast tumor models. Importantly, using our miR-155 detector platform, it was possible for the first time to visualize such miR-155 expression changes *in vivo* in a non-invasive manner. While our miR-155 detector platform reported on these changed over time in this model, further research into the precise mechanisms governing its upregulation during tumor progression is warranted, and we believe our detector platform will be an excellent tool supporting such future work.

Here, we applied this *in vivo* microRNA imaging platform in xenograft tumors models and used different mouse strains (NSG mice *versus* SCID mice) to modulate components of the immune system. To fully exploit the potential of this approach, expansion into fully immunocompetent settings will be required in the future. Therefore, additional considerations beyond appropriate microRNA controls (see above) are necessary. First, foreign components in the detector platform may elicit an immune response that can interfere with the establishment of syngeneic tumors or their progression, which will need to be appropriately controlled for. Notably, GFP and luciferase reporters were both found to be immunogenic with immune responses largely driven by the T cell compartment. Modulation of host immunity as a potential solution has been suggested and based on the concept that in transgenic mice the expression of antigens like GFP from birth onwards can induce peripheral tolerance towards GFP and luciferase expression (39, 40). This resulted in the generation of so-called ‘Glowing-Head’ mice (GH) with GFP transgene expression in anterior pituitary glands. These mouse strains were better hosts for GFP- and luciferase-expressing syngeneic tumor models than the corresponding parent mouse strains (39) with such mice now commercially available in BALB/c, C57BL/6, and FVB/N genetic backgrounds. Recently, others reported that GFP expression in extra-thymic tissues failed to fully tolerize an animal to GFP (41) and demonstrated that transgenic mice with GFP expression in dendritic cells resulted in animals that were centrally tolerized, *i.e.* mice expressing GFP in a significant proportion of thymic-antigen presenting cells. In these animals, a significantly higher number of metastases in the syngeneic 4T1 tumor setting was observed than in both BALB/c and BALB/c-GH mice. For our mircoRNA imaging approach, such tolerized mice may be one solution to overcome GFP-induced immune responses. Alternatively, one could forgo the GFP in our platform and work with NIS alone as a radionuclide reporter, albeit this would complicate cell line generation and relinquish the advantages of multi-scale imaging workflows. Second, we have used human NIS in this work (because of human cancer cells/xenograft model) but in spite of 83.6% sequence identity between human and mouse NIS orthologs (*cf.* https://www.ncbi.nlm.nih.gov/homologene), it cannot be ruled out that human NIS elicits an immune response in fully immunocompetent mice. Unfortunately, to date there are no systematic comparisons of the impact of human NIS compared to non-immunogenic mouse NIS in immunocompetent mice available. Currently, only data descriptive of both human and mouse NIS reporter function have been reported within otherwise syngeneic models; examples include adeno-associated virus tracking in cardiovascular research (42), data from vaccine research (43, 44), and some cancer models, e.g. (45). Consequently, we recommend using NIS orthologs matched to the host animal for syngeneic tumor models as a pragmatic solution until more data on the potential immunogenicity of NIS orthologs becomes available.

In conclusion, we report on the generation and characterization of a non-invasive and quantitative imaging tool intended to generate stable cell lines for the dynamic long-term *in vivo* assessment of specific microRNA expression. We provided an application example in the arena of triple-negative breast cancer research including *ex vivo* tissue analyses. Our multi-modal multi-scale detector platform developed here could also be used to answer more complex research questions, not least those which build on *ex vivo* tumor cell re-isolation with subsequent multi-parametric ‘omics’ analyses. Finally, we believe this tool is likely to prove valuable for other fields whenever researchers are interested in non-invasive quantification of spatiotemporal microRNA dynamics in animal models.

## Data availability statement

The original contributions presented in the study are included in the article/**Supplementary Material**. Further inquiries can be directed to the corresponding author.

## Ethics statement

The animal study was reviewed and approved by King’s College London Animal Welfare and Ethical Review Body and under the authority of United Kingdom Home Office PPL 70/8879.

## Author contributions

Conceptualization: GF. Data curation: AV, CL, ES, and PJ. Formal analysis: AV, CL, ES, PJ, and GF. Funding acquisition: GF. Investigation: AV, CL, ES, and PJ. Methodology: AV, ES, and FP. Project administration: GF. Supervision: GF. Validation: AV, CL, ES, and PJ. Visualization: ES, CL, and GF. Writing-original draft: GF. Writing-review and editing: all authors. All authors contributed to the article and approved the submitted version.
