# Supplementary material for: Spatiotemporal quantitative microRNA-155 imaging reports immune-mediated changes in a triple-negative breast cancer model
Source: Front Immunol. 2023 Jun 8;14:1180233. doi: 10.3389/fimmu.2023.1180233 (PMC10285160; doi:10.3389/fimmu.2023.1180233)
Supplement: Supplementary file 1 [file DataSheet_1.pdf]

Supplementary Material

Spatiotemporal quantitative microRNA-155 imaging reports immune-mediated changes in a triple-negative breast cancer model.

Elena Skourti, Alessia Volpe, Cameron Lang, Preeth Johnson, Fani Panagaki and Gilbert O. Fruhwirth

*** Correspondence:** Gilbert Fruhwirth: gilbert.fruhwirth@kcl.ac.uk

1 Supplementary Methods

Supplementary information to the generation of new DNA constructs. As microRNAs negatively regulate protein expression, we placed the reporter under the control of a repressor as an approach to enable positive correlation of obtained signals with microRNA concentrations. This was similar to a previously reported method that exploited the tetracycline repressor system for this purpose (Amendola et al., 2013). Because of a report of spontaneous loss of inducibility in some cell types following continuous growth for extended periods (Ackland-Berglund and Leib, 1995) and also to ensure flexibility for combinations of our tool with existing inducible animal models, which frequently employ tetracycline operators (Kallunki et al., 2019), we chose a different repressor system, namely the cumate-controlled operator in its repressor configuration for mammalian use (CymR; (Mullick et al., 2006)). To study cellular microRNA dynamics using the miR-on/signal-on approach *in vivo* in the long term, stable expression of the detector system in the cells of interest was required. The possibilities to achieve this were to transfer into target cells either (i) two gene expression cassettes, one for the repressor under microRNA control and another for the reporter under repressor control ('dual-vector'), or (ii) one multi-cistronic expression cassette containing all components ('single-vector').

<u>(1) microRNA detectors in the 'dual-vector' configuration:</u> *(Sensor plasmid)* An expression cassette was synthesized consisting of the EF1α promoter followed by an *XbaI* site and an open reading frame consisting of the start codon, DNA encoding an HA-tag, an eight amino acid linker (SLVRQQRP) and the CymR repressor. Right after the STOP codon, a *ClaI* site was placed followed by four repeats of matched microRNA binding sites forming the 3'UTR and a *NheI* site. This synthesized cassette (Biomatik) contained repeats of a scrambled miR sequence that was designed to not bind to any known sequence in human, mouse, or rat genomes. The cassette was supplied in pBlueScript and was subcloned using *MluI* into the iCumate-pLenti-Cloning backbone (Applied Biological Materials Inc.), thereby removing the backbone's SV40>Puromycin resistance marker. This formed the sensor plasmid for the scrambled microRNA but without a selection marker at this point (Rscr-noSel). Next, a SV40>Neomycin resistance marker was subcloned using *KpnI/XhoI* sites upstream of the EF1α promoter. The primers used to PCR amplify the SV40>Neomycin fragment are shown in Tab.S1. The final plasmid was named Rscr. To alter the 3'UTR, DNA stretches with four

repeats each of perfect matches to miR-155, miR-21 and miR-221 with flanking *ClaI/NheI* sites were synthesized as gBlocks (IDT DNA Ltd) and subcloned to produce the different sensor plasmids intended to respond to miR-155, miR-21, and miR-221 (R155, R21, R221, respectively).

(*Reporter plasmid*) To produce iCumate pLenti GFP, monomeric EGFP A206K was excised from pLNT SFFV>MCS-GFP (Volpe et al., 2018) using *ScaI* and *XhoI* restriction enzymes and subcloned into the commercially available iCumate pLenti Cloning lentiviral vector (Applied Biological Materials Inc.). Human NIS (hNIS) was then excised from pLNT SFFV>hNIS-RFP using *BamHI* and *KpnI* sites and subcloned into iCumate pLenti GFP to generate the reporter plasmid iCumate pLenti hNIS-GFP.

(2) microRNA detectors in the 'single-vector' configuration: The completed sensor plasmids of the dual-vector system served as the starting material. Each of them was modified to become the unidirectional bicistronic vector through subcloning of the CMV/CuO>NIS-GFP part of the reporter vector (using *NdeI* and *NotI* sites). MicroRNA-specificity was stemming from the sensor plasmids serving as starting material.

(3) Lentiviral overexpression plasmids for the mircoRNAs corresponding to the 3'UTRs were ordered from Biosettia (pLV miR-155, pLV miR-21, pLV miR-221 and pLV miR-scr) and used as supplied with their RFP-blasticidine resistance markers.

(4) MiR-locker plasmids were also ordered from Biosettia. As they were only available in GFP-blasticidin configurations, they were consequently modified by replacing the GFP component of GFP-blasticidin with RFP from the pLV miR-scr plasmid (subcloning of RFP to replace GFP using the *XhoI* sites and the primer pair listed in Supplementary Table S1).

Supplementary information to lentivirus production and cell transduction. Lentiviral particles were generated in HEK293T cells using the indicated transgene plasmids, together with the 'packaging plasmids' pCMV ΔR8.91 and pCMV VSV-G (conferred tropism to virus particles for human and rodent cell lines). The plasmids were transfected into the producer cells using the linear polyethylenimine method. Therefore, the plasmids (total amount of 7.5 µg DNA) were mixed at a mass ratio of 4 (transgene) : 3 (Δ8.91) : 1 (VSV-G) and then diluted into 500 µL DMEM, which was followed by the addition of a 3-fold mass excess of PEI (in relation to plasmid DNA, 22.5 µg; PEI was sourced from Polysciences) and 15 min incubation at room temperature. Producer cells received fresh growth medium (80 µL/cm^2) onto which the transfection mix was slowly dropped with a pipet. Lentiviral supernatants were collected 48 h post-transfection, filtered through a 0.45 µm pore filter. Virus solutions were used freshly to transduce cells. Cells were selected according to the resistance marker on the relevant transgene plasmid. Cell selection started 24h post transduction and was performed using either neomycin (2 mg/mL), puromycin (2 µg/mL) for detector cells and/or blasticidin 4 µg/mL) for cell lines stably overexpressing microRNAs or miR-lockers.

Supplementary information to CellProfiler use. A segmentation workflow example is shown in Fig.S10.

Supplementary Figures and Tables

1.1 Supplementary Tables

Supplementary Tab.S1. Primers for the generation of DNA constructs.

Construct	Primer name	Sequence
Sensor plasmid	KpnI SV40 FWD	5′-CGAGGTACCGGTGTGGAAAGTCCCCAGGCTCCCCAGGCAGGC-3′
	XhoI NeoR REV	5′-CGTCTCGAGTCAGAAGAACTCGTCAAGAAGGCGATAGAAGGC-3′
MiR-locker plasmids	RFP XhoI FWD	5′-GACGCTAGCTCGAGCTTTTGGAGTAC-3′
	RFP XhoI REV	5′-AAGGTACCGAGCTCGAGAATTCCAGGCGGGGAGGC-3′

1.2 Supplementary Figures

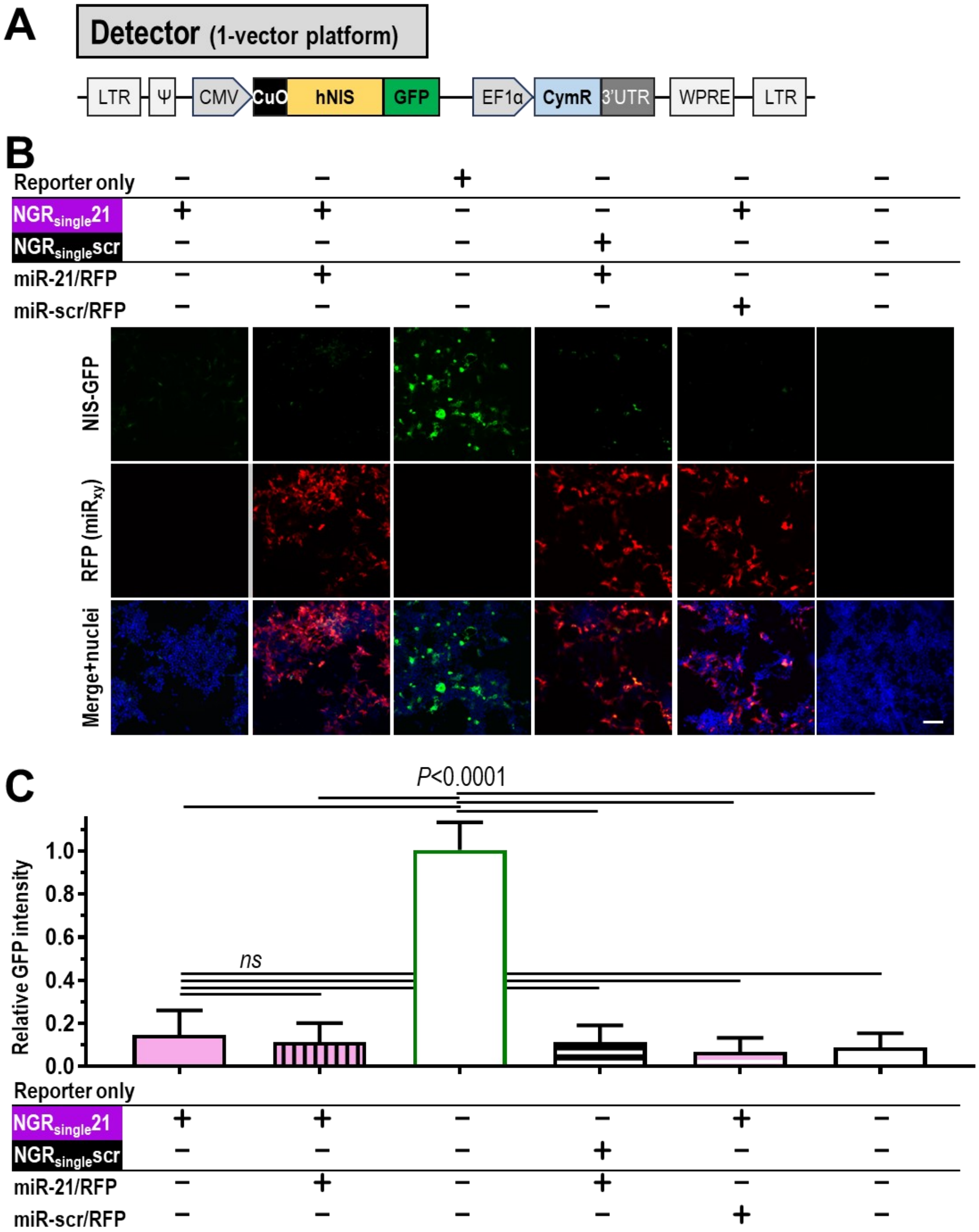

Supplementary Fig.S1. Construct design and validation of the microRNA detector employing 'single-vector' platform approach. | (A) Scheme of the multi-cistronic construct. For details to the individual components, see Fig.1A. **(B)** Validation of the detector principle in HEK293T cells using the single-vector platform. HEK293T cells were chosen as they express hardly any miR-21 (Yamamichi et al., 2009;Ribas et al., 2012;Tian et al., 2012). The sensor is either under the control of miR-21 (purple, $NGR_{single}21$) or a non-targeting scrambled microRNA ($NGR_{single}scr$, black). For validation, microRNAs (miR-21 or miR-scr) were co-expressed from separate plasmids (detectable by RFP co-expression) in the indicated samples. Typical results from the same experimental batch are

shown. Scale bar is 100µm across all images. **(C)** Cumulative results of (B) with bars belonging to samples as indicated in (B) with $n \geq 3$ independent experiments. Error bars represent SD; statistical analysis by one-way ANOVA with Tukey's multiple comparison correction. Relevant non-significant comparisons are shown alongside all significant differences. These results demonstrated that the single-vector approach repressed the reporter as desired but failed to switch reporter expression on upon microRNA co-expression (Fig.S1), while the 'dual-vector' approach performed well (Fig.1). Our results agree with a previous study that had employed fluorescent protein reporters in combination with the well-established tetracycline repressor. The authors had also compared the performance of a 'single-vector' with a different 'dual-vector' design in their system, and they had obtained similar results to us: their 'dual-vector' approach performed well, while their 'single-vector' experiments were limited, in their case by leakiness and oscillatory behavior (Amendola et al., 2013). They had used the strong spleen focus forming virus (SFFV) promoter to drive repressor expression and had employed a bidirectional promoter design, which might have directly contributed to the observed oscillatory behavior. Hence, we adopted a weaker mammalian EF1α promotor to drive repressor expression as part of a sequential unidirectional design, but also our 'single-vector' approach did not result in reliable and sensitive microRNA detection. Another study had employed the simian virus 40 promoter together with a cumate switch repressor, but the authors did not report the generation of stable cell lines (Ezzine et al., 2013), which explained why they did not observe these issues that appear to be intrinsic to the 'single-vector' design.

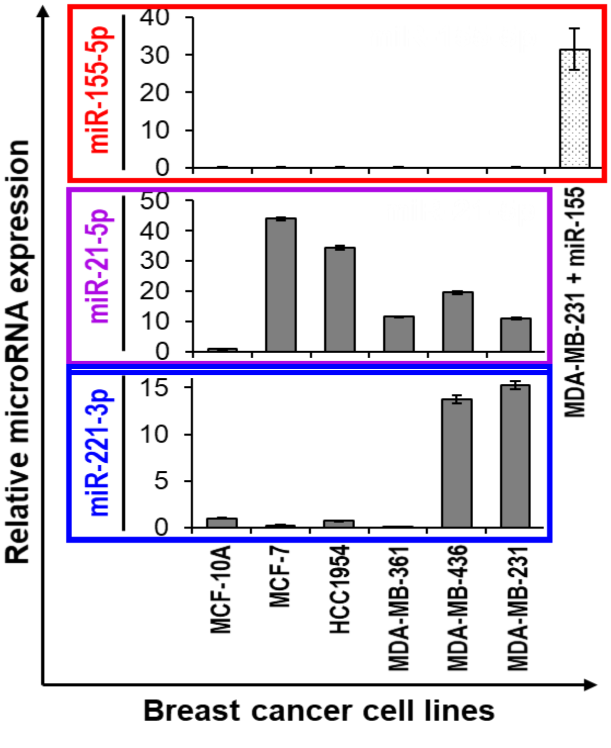

Supplementary Fig.S2. Comparative expression of miR-21, miR-155, and miR-221 in a panel of human breast cancer cell lines. | The microRNAs miR-21, miR-221 and miR-155 have previously been implicated in breast cancer progression (Yan et al., 2008;Mattiske et al., 2012;Dinami et al., 2014;Ye et al., 2014;Zhao et al., 2015;Pan et al., 2016) and we validated their expression by quantitative RT-PCR here. Cumulative data is shown of n=4 determinations from independent cell batches. (*Top/red*) In none of the investigated parental cell lines we detected miR-155 expression. As a positive control, MDA-MB-231 cells were transduced with lentiviruses to transfer an expression

cassette for overexpression of miR-155, which was significantly different to all other samples; $P<0.0001$). (*Middle/purple*) MiR-21 was expressed significantly higher in all cancer cell lines when compared to MCF-10A cells ($P<0.001$ for all comparisons). Moreover, except for the comparison of MDA-MB-361 with MDA-MB-231, which was not showing significant differences, all other comparisons between cancer cells were significant ($P<0.0001$). *(Bottom/blue)* MiR-221 was significantly higher expressed in the two triple-negative breast cancer cell lines MDA-MB-231 and MDA-MB-436 ($P<0.001$ for both compared to all other cell lines), while there were no significant differences between the other cell lines. All data were normalized to the pre-cancerous cell model MCF-10A. Note that y-axis scales differ between different microRNA panels. Error bars represent SD; statistical tests were performed using one-way ANOVA using Tukey's multiple comparison correction.

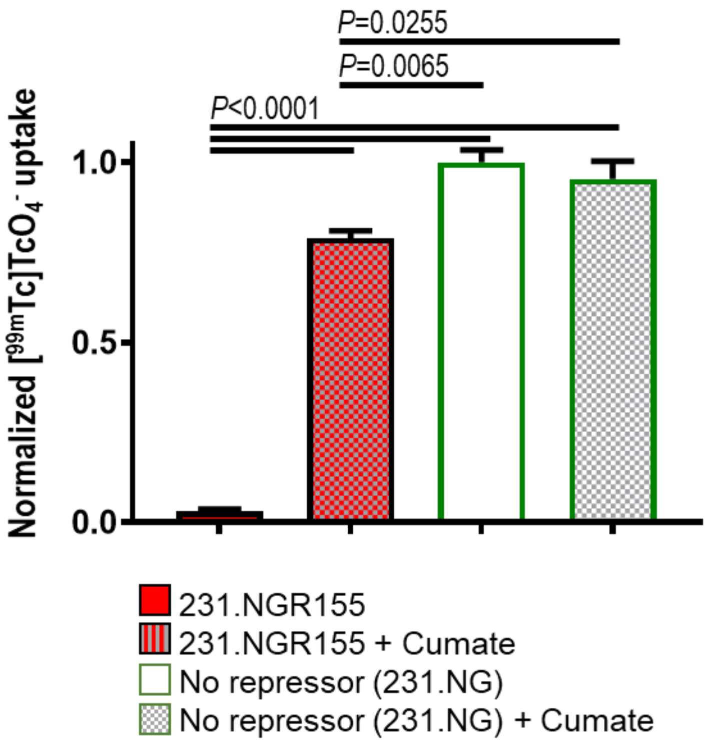

Supplementary Fig.S3. Response to cumate in MDA-MB-231 cells stably expressing the miR-155 detector. | NIS-GFP reporter signals analyzed by radiotracer $[^{99m}Tc]TcO_4^-$ uptake as a read-out for NIS reporter function. In 231.NGR155 cells treated with cumate, the signal repression was pharmacologically released despite the absence of the miR-155. This was as expected and in line with the predicted function of this repressor system (Mullick et al., 2006). Data are normalized to maximum uptake in repressor-free stable MDA-MB-231 cells only expressing the reporter (231.NG). Cumulative data of n=3 independent experiments with error bars representing SD. Relevant statistical comparison shown (calculated by one-way ANOVA using Tukey's multiple comparison correction).

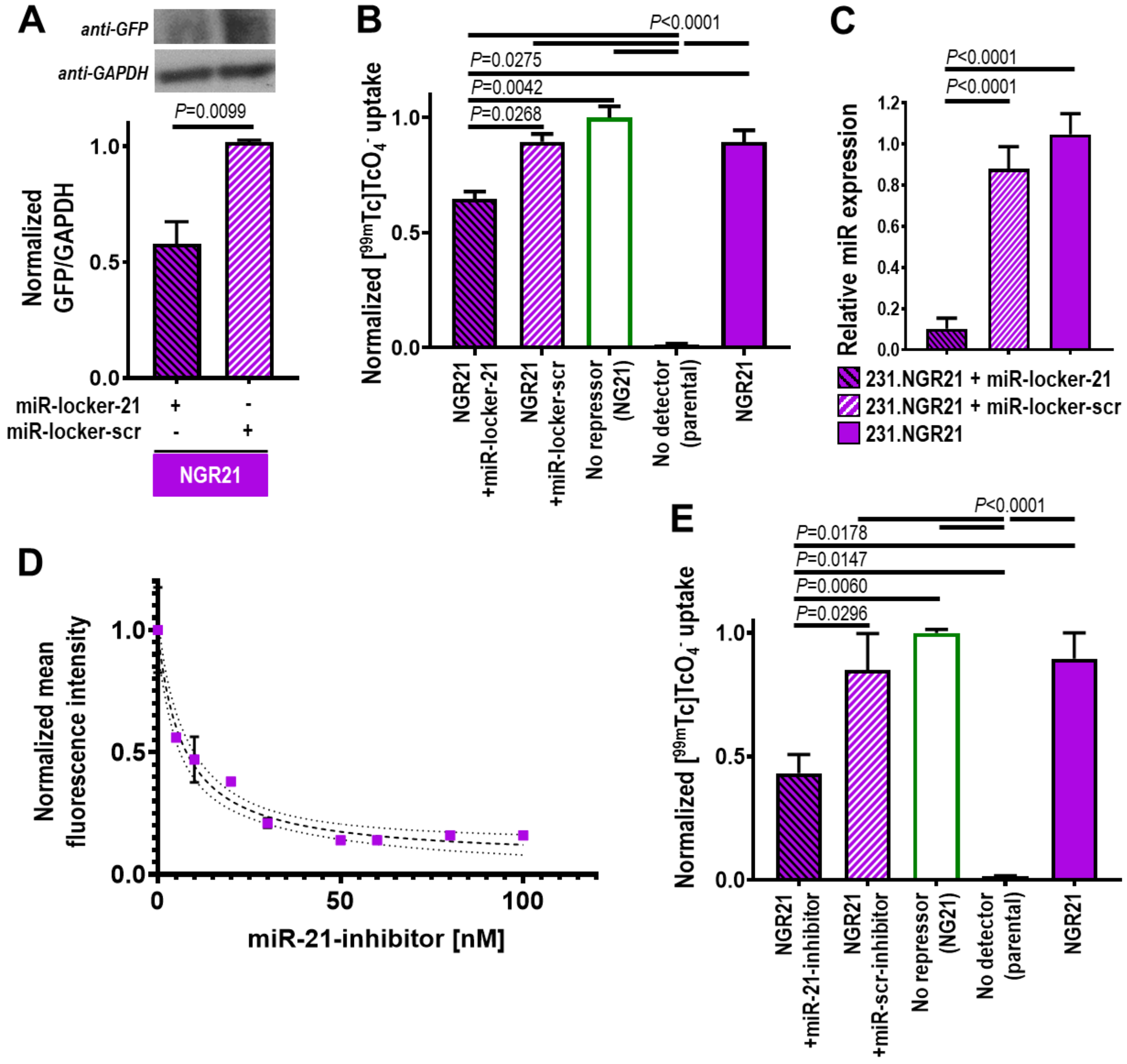

Supplementary Fig.S4. Detection of miR-21 modulation in stable 231.NGR21 cells. | 231.NG21 cells show NIS-GFP signals under untreated conditions due to the presence of endogenous levels of miR-21 (cf. Fig.2 and Fig.S2). Partial reduction of endogenous miR-21 levels in MDA-MB-231 cells stably expressing the miR-21 detector was achieved using either co-expressed antagonistic miR-21 (miR-21-locker) or by treatment of these cells with a miR-21 inhibitor. **(A)** Immunoblotting reveals a reduction of NIS-GFP signals upon co-expression of the specific antagonistic miR-21-locker but not the unspecific miR-scr-locker. Representative immunoblot for GFP with anti-GAPDH as a loading control is shown at the top with the graph below showing cumulative data of *n*=3 independent experiments. The *P*-value was calculated using a Student's *t*-test. **(B)** NIS-GFP reporter signals were quantified though $[^{99m}Tc]TcO_4^-$ uptake as a read-out for NIS reporter function. The specific antagonistic miR-21-locker significantly reduced radiotracer uptake compared to treatment with either the unspecific miR-scr-locker or compared to untreated 231.NGR21 cells. Shown are cumulative data of *n*=3 independent experiments with error bars representing SD. *P*-values were calculated by one-way ANOVA using Tukey's multiple comparison correction. **(C)** Cumulative quantitative RT-PCR analysis of miR-21 of indicated cell lines used in (A) and (B). Data of *n*=3 independent experiments are shown with error bars representing SD. *P*-values were calculated by one-way ANOVA using Tukey's multiple

comparison correction. **(D)** GFP fluorescence intensity of 231.NGR21 cells decreased upon treatment with increasing amounts of the miR-21 inhibitor. Dose-dependency was fit using a three-parameter dose-response model, which revealed an IC_{50} for the miR-21-inhibitor of 7.0 nM (fitted curve shown as dashed line with dotted lines delineating the 95% confidence intervals; 95% confidence interval of IC_{50} is [4.6;10.7] nM); *n*=3 with error bars representing SD. **(E)** Radiotracer uptake assay as in (B) but with cells treated with 50nM of the indicated miR-inhibitors. Shown are cumulative data of *n*=3 independent experiments with error bars representing SD. *P*-values were calculated by one-way ANOVA using Tukey's multiple comparison correction. Generally, most relevant *P*-values have been added into graph panels. In (B) and (E), the *P*-values for all comparisons with the "no detector/parental cell" (black frame) conditions were *P*<0.0001 and no significant differences were found between the "no repressor/NG21 cell" (green frame) conditions and both NGR21+miR-locker-scr/miR-scr-inhibitor and NGR21 samples. "No repressor/NG21 cell" (green frame) conditions compared to NGR21+miR-locker-21/miR-21-inhibitor in those panels were *P*=0.0042 and *P*=0.0060, respectively.

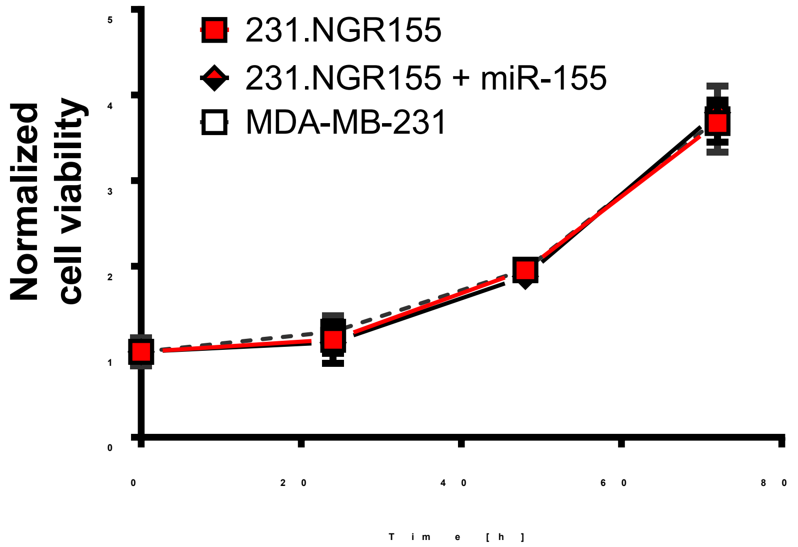

Supplementary Fig.S5. Proliferation assay comparing various stable microRNA detector and control cell lines. | Cell proliferation was analyzed using the Alamar Blue fluorescence assay and data were normalized to the beginning of the experiment. Alamar Blue fluorescence was linearly dependent on cell numbers under the used conditions and obtained fluorescence signals report on time-dependent increases in cell number. Cumulative data from *n*≥3 different experiments are shown with error bars representing SD. No significant differences between indicated cells were found at any time point.

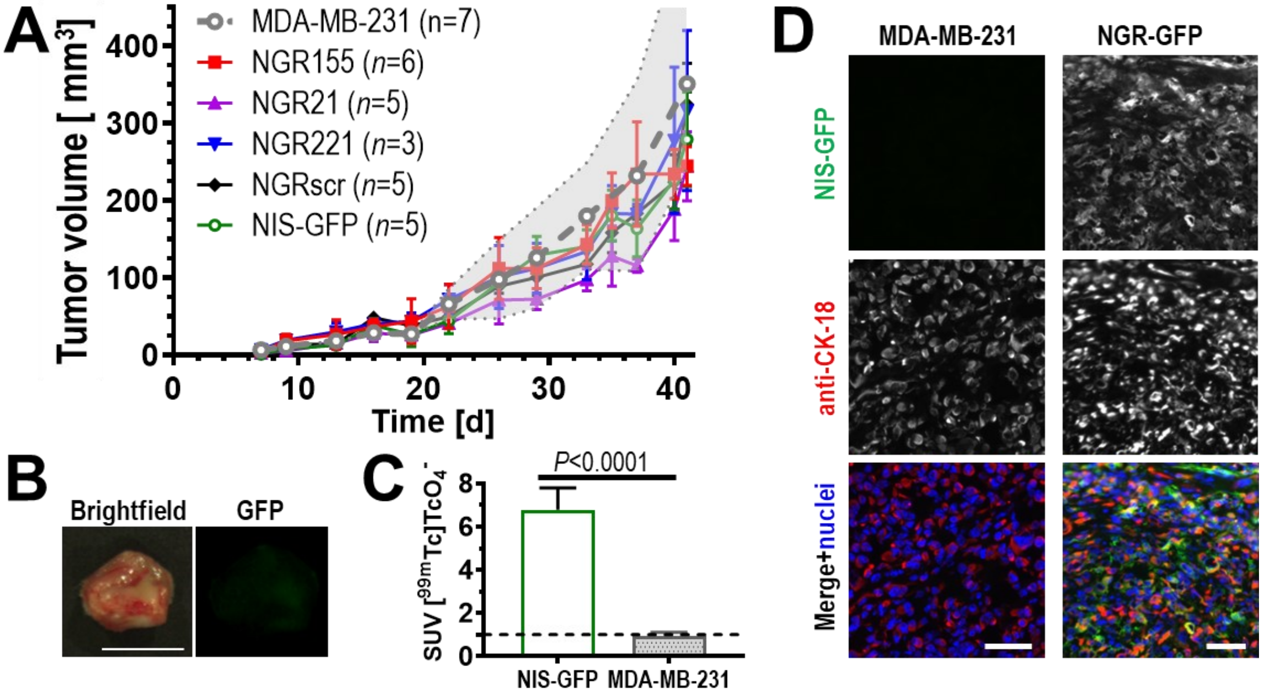

Supplementary Fig.S6. Typical data from tumors established from the parental MDA-MB-231 cell line. | (A) MDA-MB-231 tumor growth compared to microRNA detector expressing tumor models also shown in Fig.4B. Cumulative data of $n = 7$ MDA-MB-231 tumors are shown with shading indicating ±SD. Repeated measures mixed-effect analysis including Geisser-Greenhouse correction comparing simple effect within time points using Tukey's multiple comparison correction revealed no significant differences to other tumor cohorts ($P > 0.05$). **(B)** A typical example of a harvested MDA-MB-231 tumor is shown. The brightfield image was taken under room light, while the fluorescence image was taken using blue light LED excitation and through a green light filter in the dark using the same settings as in Fig.4; the scale bar is 1 cm. **(C)** Cumulative data of tumor radioactivity ($n = 5$ for 231.NG and $n = 4$ for MDA-MB-231 tumors); error bars=SD. Unpaired Student's *t*-test revealed a significant difference between tumors established from reporter-positive or parental MDA-MB-231 cells. **(D)** Typical immunofluorescence histology micrographs of indicated tumor sections that were stained for human CK-18. In merged images, anti-CK-18 staining is pseudocolored in red, NIS-GFP in green, and nuclei in blue; scale bars are 50 µm.

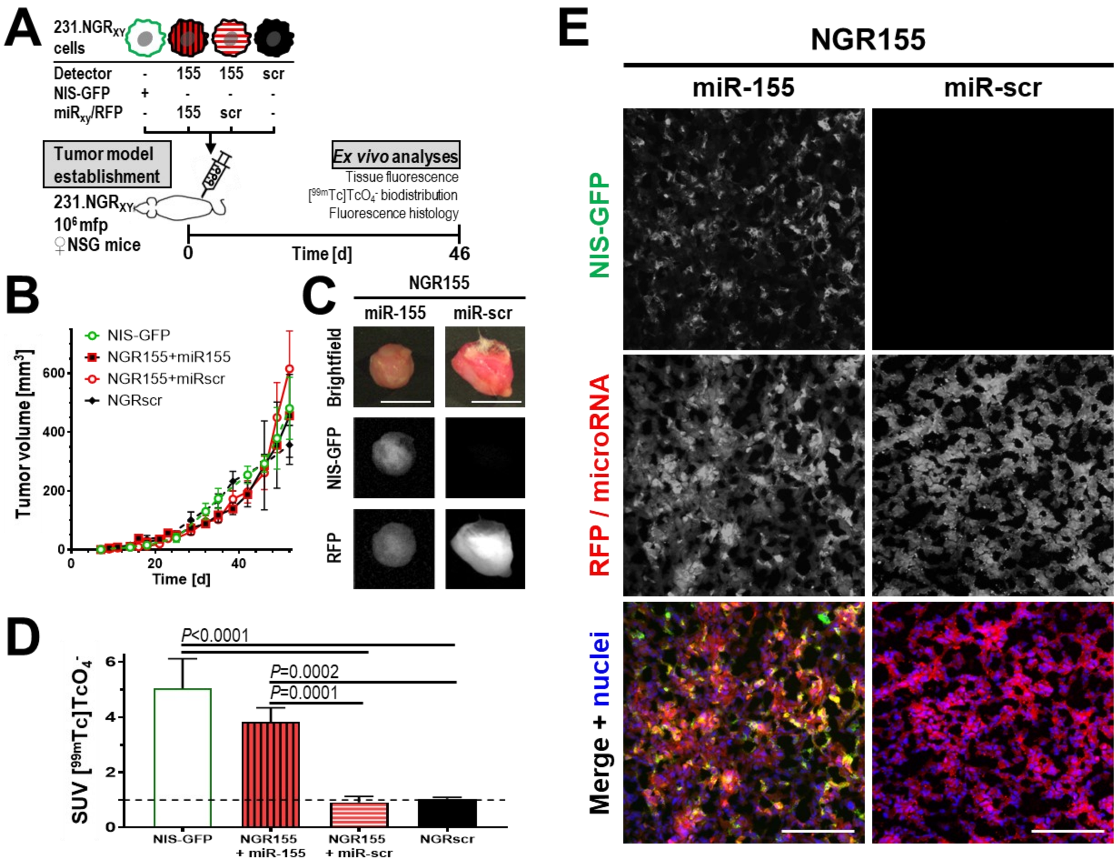

Supplementary Fig.S7. *In vivo* validation of miR-155 detector expressing MDA-MB-231 cell lines in immunodeficient NSG mice. | (A) Experimental scheme. **(B)** Tumor growth curves of the two indicated tumor models. Cumulative data (n=4 mice per cohort) are shown with error bars representing SD. Repeated measures mixed-effect analysis including Geisser-Greenhouse correction comparing simple effect within time points using Tukey's multiple comparison correction revealed no significant differences between tumor cohorts (P>0.05). On day 40, animals received 20 MBq of the NIS radiotracer $[^{99m}Tc]TcO_4^-$ and were culled 45 min later with tumors harvested and analyzed immediately for their tissue fluorescence and tissue radioactivity by γ-counting. **(C)** Typical examples of harvested tumors are shown. (*top*) Brightfield images were taken under room light, while fluorescence images were taken in the dark using either (*middle*) blue light excitation paired with a green emission filter for analysis of NIS-GFP or (bottom) green light excitation paired with a red emission filter to analyze RFP; scale bars are 1 cm. **(D)** Cumulative data of tumor radioactivity (n=4 per cohort); error bars are SD. SUV > 1 is indicative of relevant tissue uptake. One-way ANOVA revealed NGR155 tumors co-expressing miR-155 showed significantly higher NIS-GFP expression than both NGR155 tumors co-expressing the control miR-scr and NGRscr control tumors. **(E)** Typical fluorescence histology micrographs of tumor sections cut from tumors in (A). In merged images, cells expressing RFP alongside the indicated microRNAs are pseudocolored in red, NIS-GFP expressing cells pseudocolored in green, and nuclei in blue; scale bares are 100 µm. RFP signals (in B and D) demonstrating miR presence in both tumor groups. These results are in line with our previous observations in cell lines, *i.e.* NIS-GFP signals upon miR-155 overexpression and no signals upon miR-scr overexpression Fig.S3) as shown by tissue analyses (fluorescence and radioactivity analysis of whole tumors and histology).

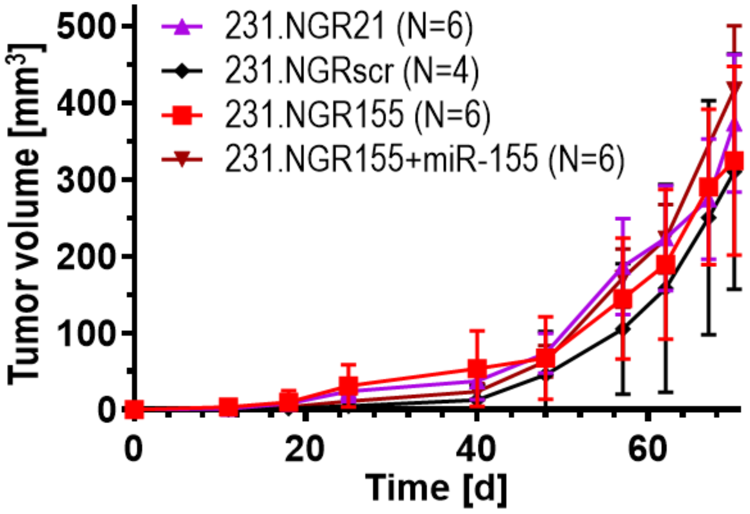

Supplementary Fig.S8. *In vivo* growth of tumors established from indicated microRNA detector cell lines in immunocompromised SCID mice. | Data are supplementary to Fig.5-6. Cumulative data (for *n* see legend) are shown with error bars representing SD. Repeated measures mixed-effect analysis including Geisser-Greenhouse correction comparing simple effect within time points using Tukey's multiple comparison correction revealed no significant differences between tumor cohorts (P>0.05).

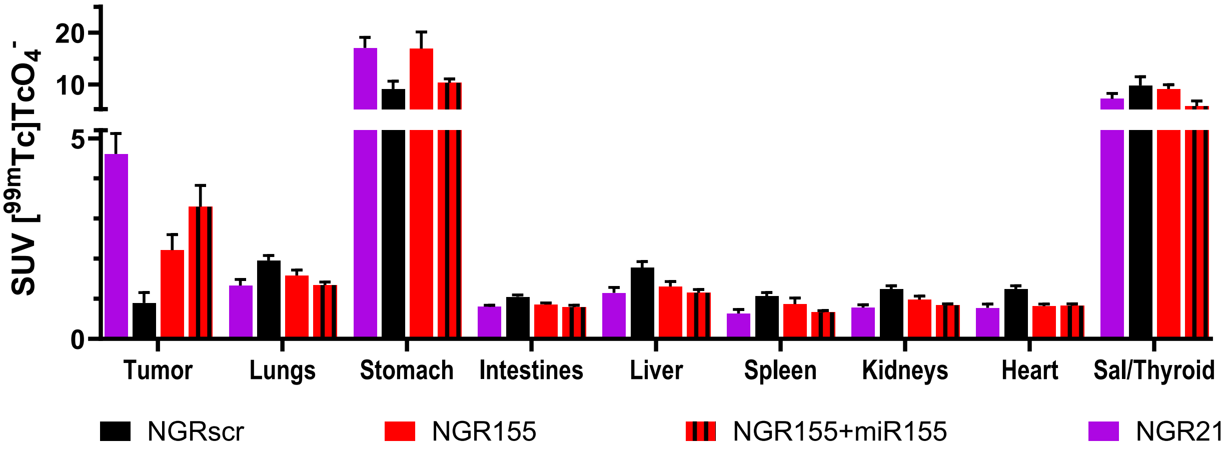

Supplementary Fig.S9. Additional ex vivo γ-counting data from other organs than the tumor and supplementing Fig.6A. | Cumulative data with error bars representing SE; *n*=6 for all but NGRscr tumors for which *n*=4. For relevant statistical analyses from tumor tissues see Fig.5A. Large error bars in the endogenously NIS expressing stomach are expected and the differences may depend on the nutritional activity of the mice. Other tissues did not show any statistically significant differences between animal cohorts.

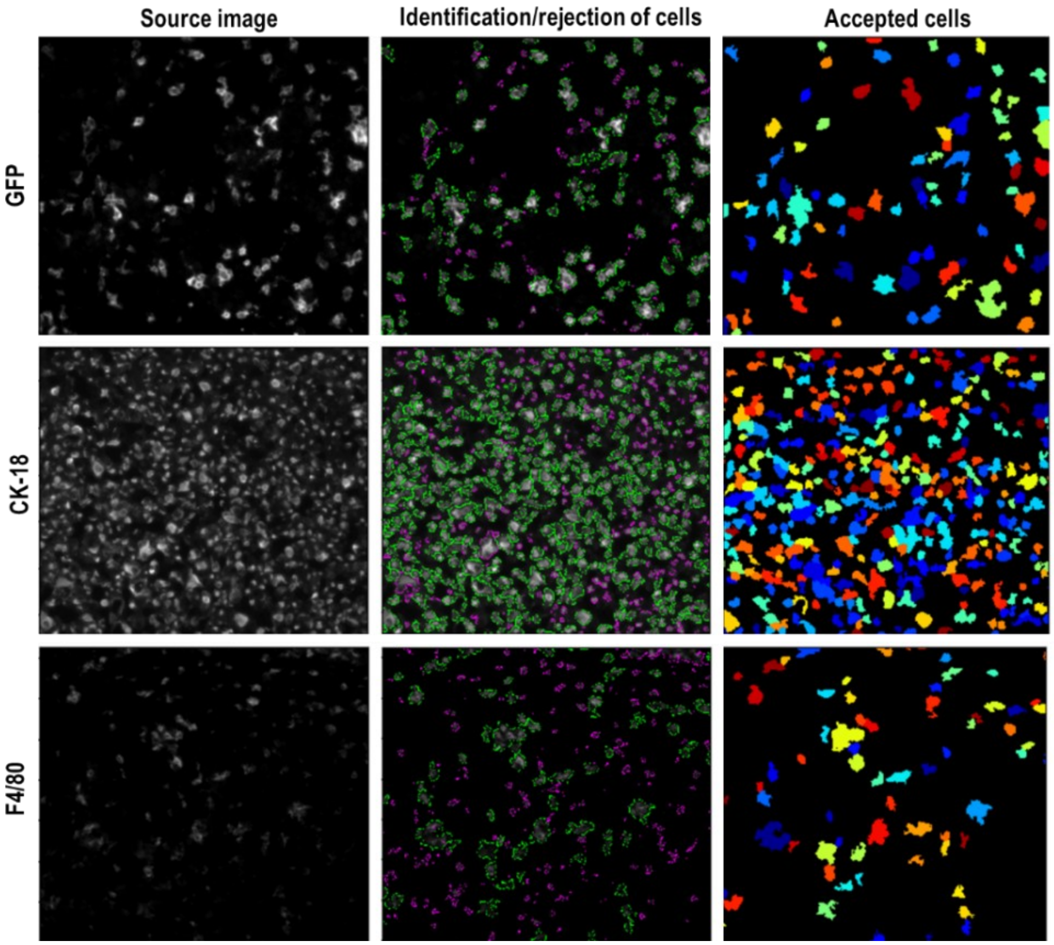

Supplementary Fig.S10. Scheme showing the workflow of the CellProfiler-assisted automated cell segmentation. | The purpose is to calculate NIS-GFP^{+}CK-18^{+} or F4/80^{+} cell percentages in tissues. *(left)* Raw fluorescence intensity images of indicated markers. *(middle)* Green are accepted objects, purple rejected objects. *(right)* accepted objects individually colored to identify them as separate objects.

2 References used in Supplement

Ackland-Berglund, C.E., and Leib, D.A. (1995). Efficacy of tetracycline-controlled gene expression is influenced by cell type. *Biotechniques* 18**,** 196-200.

Amendola, M., Giustacchini, A., Gentner, B., and Naldini, L. (2013). A double-switch vector system positively regulates transgene expression by endogenous microRNA expression (miR-ON vector). *Mol Ther* 21**,** 934-946.

Dinami, R., Ercolani, C., Petti, E., Piazza, S., Ciani, Y., Sestito, R., Sacconi, A., Biagioni, F., Le Sage, C., Agami, R., Benetti, R., Mottolese, M., Schneider, C., Blandino, G., and Schoeftner,

S. (2014). miR-155 drives telomere fragility in human breast cancer by targeting TRF1. *Cancer Res* 74, 4145-4156.

Ezzine, S., Vassaux, G., Pitard, B., Barteau, B., Malinge, J.M., Midoux, P., Pichon, C., and Baril, P. (2013). RILES, a novel method for temporal analysis of the in vivo regulation of miRNA expression. *Nucleic Acids Res* 41, e192.

Kallunki, T., Barisic, M., Jaattela, M., and Liu, B. (2019). How to Choose the Right Inducible Gene Expression System for Mammalian Studies? *Cells* 8.

Mattiske, S., Suetani, R.J., Neilsen, P.M., and Callen, D.F. (2012). The oncogenic role of miR-155 in breast cancer. *Cancer Epidemiol Biomarkers Prev* 21, 1236-1243.

Mullick, A., Xu, Y., Warren, R., Koutroumanis, M., Guilbault, C., Broussau, S., Malenfant, F., Bourget, L., Lamoureux, L., Lo, R., Caron, A.W., Pilotte, A., and Massie, B. (2006). The cumate gene-switch: a system for regulated expression in mammalian cells. *BMC Biotechnol* 6, 43.

Pan, Y., Li, J., Zhang, Y., Wang, N., Liang, H., Liu, Y., Zhang, C.Y., Zen, K., and Gu, H. (2016). Slug-upregulated miR-221 promotes breast cancer progression through suppressing E-cadherin expression. *Sci Rep* 6, 25798.

Ribas, J., Ni, X., Castanares, M., Liu, M.M., Esopi, D., Yegnasubramanian, S., Rodriguez, R., Mendell, J.T., and Lupold, S.E. (2012). A novel source for miR-21 expression through the alternative polyadenylation of VMP1 gene transcripts. *Nucleic Acids Res* 40, 6821-6833.

Tian, W., Dong, X., Liu, X., Wang, G., Dong, Z., Shen, W., Zheng, G., Lu, J., Chen, J., Wang, Y., Wu, Z., and Wu, X. (2012). High-throughput functional microRNAs profiling by recombinant AAV-based microRNA sensor arrays. *PLoS One* 7, e29551.

Volpe, A., Man, F., Lim, L., Khoshnevisan, A., Blower, J., Blower, P.J., and Fruhwirth, G.O. (2018). Radionuclide-fluorescence Reporter Gene Imaging to Track Tumor Progression in Rodent Tumor Models. *J Vis Exp* 133, e57088.

Yamamichi, N., Shimomura, R., Inada, K., Sakurai, K., Haraguchi, T., Ozaki, Y., Fujita, S., Mizutani, T., Furukawa, C., Fujishiro, M., Ichinose, M., Shiogama, K., Tsutsumi, Y., Omata, M., and Iba, H. (2009). Locked nucleic acid in situ hybridization analysis of miR-21 expression during colorectal cancer development. *Clin Cancer Res* 15, 4009-4016.

Yan, L.X., Huang, X.F., Shao, Q., Huang, M.Y., Deng, L., Wu, Q.L., Zeng, Y.X., and Shao, J.Y. (2008). MicroRNA miR-21 overexpression in human breast cancer is associated with advanced clinical stage, lymph node metastasis and patient poor prognosis. *RNA* 14, 2348-2360.

Ye, X., Bai, W., Zhu, H., Zhang, X., Chen, Y., Wang, L., Yang, A., Zhao, J., and Jia, L. (2014). MiR-221 promotes trastuzumab-resistance and metastasis in HER2-positive breast cancers by targeting PTEN. *BMB Rep* 47, 268-273.

Zhao, Y., Zhao, L., Ischenko, I., Bao, Q., Schwarz, B., Niess, H., Wang, Y., Renner, A., Mysliwietz, J., Jauch, K.W., Nelson, P.J., Ellwart, J.W., Bruns, C.J., and Camaj, P. (2015). Antisense inhibition of microRNA-21 and microRNA-221 in tumor-initiating stem-like cells modulates tumorigenesis, metastasis, and chemotherapy resistance in pancreatic cancer. *Target Oncol* 10, 535-548.

***** End of Supplementary Information *****
